# Parametrization of Zirconium for DFTB3/3OB: A Pathway to Study Complex Zr‐Compounds for Biomedical and Material Science Applications

**DOI:** 10.1002/jcc.70140

**Published:** 2025-05-26

**Authors:** Armin Penz, Jakob Gamper, Josef M. Gallmetzer, Felix R. S. Purtscher, Thomas S. Hofer

**Affiliations:** ^1^ Department of General, Inorganic and Theoretical Chemistry University of Innsbruck Innsbruck Austria

**Keywords:** DFTB3, 3OB, density functional tight binding, metal‐organic framework, zirconium

## Abstract

This work presents the extension of the semi‐empirical density functional tight binding method, DFTB3, to include zirconium for biomedical and material science applications. The parametrization of Zr has been carried out in consistency with already established 3OB parameters including the elements C, H, N, O, S, P, Mg, Zn, Na, K, Ca, F, Cl, Br, and I. Zirconium‐ligand association and reaction energies have been compared with results from quantum chemical calculations obtained at MP2 and DFT (PBE and B3LYP) level of theory, as well as with those from the semi‐empirical methods DFTB2/PTBP and GFN2‐xTB. A structural validation has been carried out on 1,897 compounds reported in the Cambridge Structural Database, revealing an average root mean square deviation comparable to that obtained at semi‐empirical (DFTB2/PTBP and GFN2‐xTB) level of theory and via the novel neural network potential MACE‐MP‐0. To provide a critical validation of the newly derived parameters, the structure of the biomedically relevant Zr‐DFO complex has been evaluated with respect to a DFT (B3LYP) reference calculation. In addition, extensive DFTB3 MD simulations of the two prominent metal‐organic frameworks UiO‐66 and UiO‐67 have been performed. The results demonstrate the applicability of the newly developed parameters for the study of zirconium‐containing metal‐organic frameworks, when compared to experimental measurements, as well as computational approaches.

## Graphical abstract

Zirconium is of significant interest in the domain of material science and biomedical applications. The development of fast and accurate computational methodologies is of paramount importance for studying the properties of these materials. This article presents an extension of the well‐established DFTB3/3OB approach to Zr, with the objective of facilitating such studies.

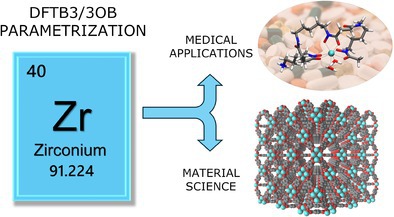

**Abbreviations**
ASE: atomic simulation environment
CSD: cambridge structural database
DFO: desferrioxamine B
DFT: density functional theory
DFTB: density functional tight binding
KS: Kohn‐Sham
MACE: multilayer atomic cluster expansion
MAD: mean absolute deviation
MAX: maximum absolute deviation
MD: molecular dynamics
MOF: metal‐organic framework
PBE: Perdew‐Burke‐Ernzerhof
PTBP: periodic table baseline parameters
RMSD: root mean square deviation
SBU: secondary building unit
SCC: self‐consistent charge
xTB: extended tight binding
ZPE: zero‐point energy

## 1 Introduction

The fifth period early transition metal zirconium is known for its wide variety of applications. 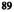
Zr radiolabeled complexes have been successfully employed to track cell migration [1], study the inflammatory immune response [2] and as oncological radiopharmaceuticals to locate and characterize tumor lesions [3]. Its alloys are utilized in orthopedic and dental applications due to their elevated mechanical stability, corrosion resistance, compatibility with magnetic resonance imaging, and bioactivity through a bone‐like apatite layer at the surface level [4]. Zirconium alloys are also utilized in the structural materials of nuclear power plants for fuel assemblies and cladding for pressure pipes and thermal reactors [5]. 2d‐layered α‐zirconium phosphate is a promising anticancer drug delivery nanovehicle and supporting material for the oxygen evolution reaction [6]. Zirconium salts are versatile catalysts in organic chemistry, including the synthesis of heterocycles, carbon‐carbon bond formation, and multicomponent reactions [7]. In addition to those, recent applications feature Zr as part of metal‐organic framework materials [8, 9, 10]. Metal‐organic frameworks (MOFs) [11] are a class of nanoporous materials that have attracted significant attention over the past decades due to their extensive applications in gas adsorption and separation [12], chemical sensing [13], heterogeneous catalysis [14] and biomedical drug delivery [15]. Their physico‐chemical properties are highly tunable by the choice of inorganic nodes and organic linkers, allowing the design of materials with properties that are tailored to specific applications. Although novel MOF materials are being reported at an increasing rate [16], most of them show limited chemical, thermal, hydrothermal, and mechanical stability in comparison to zeolites [17]. This limitation drastically restricts their potential for industrial utilization and prompts the necessity for the development of new MOFs with enhanced stability under increased temperature and solvent conditions.

In 2008, Lillerud et al. [18] pioneered the synthesis of zirconium‐based metal‐organic frameworks by introducing the *Universitetet i Oslo* (UiO) MOF class. These porous materials demonstrate an exceptional surface area, an elevated decomposition temperature, and high chemical stability, along with tolerance to water and other solvents. The remarkable properties of these frameworks are attributed to the inorganic node, which consists of a Zr
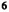
O_4_(OH)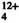 cluster. This cluster exhibits strong zirconium‐oxygen bonds and enables the coordination of up to 12 linker molecules. In addition, zirconium has been found to be present in numerous biological systems [19]. It exhibits low toxicity and is capable of being readily eliminated by the human body [20, 21]. This stability and biocompatibility paves the way for Zr‐based MOFs in biological applications, such as serving as nanoscale drug delivery devices and electrochemical biosensing of drugs, biomarkers, pesticides and food additives [22, 23, 24, 25, 26, 27, 28, 29, 30, 31]. Post‐Hartree‐Fock and density functional theory (DFT) approaches are frequently employed to access the properties of these materials on a computational basis [32, 33, 34, 35, 36, 37]. Nevertheless, the utilization of these methods incurs unfeasible computational demand with increasing system size and within the regime of molecular dynamics simulations of complex chemical systems, thereby rendering force field approaches a prevalent substitute for the treatment of metal‐organic frameworks [38, 39, 40, 41, 42]. Despite the perception of force fields as expeditious, cost‐effective, and scalable methods to accurately describe pristine MOFs, they demonstrate limited accuracy in determining host‐guest interactions and treating open metal sites of certain MOF systems. These issues can be tackled by employing semi‐empirical quantum mechanical methods. The fast, robust and accurate DFTB3 [43] method employing the 3OB [44] set of parameters has previously been established for the investigation of various MOFs, as well as guest@MOF systems [45, 46, 47, 48, 49]. Several additional DFTB extensions for the 3OB parameter set have been reported for transition and alkali metals such as Li [50], Ti [51], Ni [52], Cu [53] and Pt [54]. However, to the best of our knowledge, no third‐order DFTB extension involving Zr has been developed as of yet. This work aims to fill this methodological gap by augmenting the 3OB parameter set for the use of zirconium‐containing complexes and materials, with particular emphasis on metal‐organic frameworks and medicinally relevant complexes. The latter will be substantiated through a case study of the complex reagent zirconium desferrioxamine B (Zr‐DFO), which holds great significance as a radiotracer in immuno positron emission tomography (PET) [55, 56, 57]. In the upcoming section, the DFTB3 approach is briefly reviewed, followed by a detailed description of the parametrization process for zirconium. The subsequent validation of the parametrization is then achieved through the calculation of association and reaction energies of numerous complexes, the prediction of structures from the Cambridge Structural Database (CSD) and the complex Zr‐DFO, as well as extensive molecular dynamics (MD) simulations of the two prominent MOFs UiO‐66 and UiO‐67. In the final section, a synthesis of the results of this study is presented, followed by a discussion of the conclusions that can be drawn from them.

## 2 Concise Review of DFTB3

The parametrization of zirconium serves to augment the existing 3OB set of parameters, which currently comprises the elements C, H, N, O, S, P, Mg, Zn, Na, K, Ca, F, Cl, Br, and I [44, 58, 59, 60]. In the following, a brief revision of the DFTB3 method is given, illustrating the role of the adjustable parameters in determining the total energy. For a comprehensive derivation of the equations please refer to the original DFTB1 [61], DFTB2 [62], and DFTB3 [43] publications, as well as more recent reviews [63, 64]. Atomic units have been used in the expression of all the following equations and expressions where applicable.

In DFT, the electron density ρ(**r**) is determined by minimizing the total energy. DFTB commences with the description of ρ(**r**) via a reference density $\rho_{0}$(**r**) plus a density fluctuation term δρ(**r**), as shown in Equation (1).

(1) $$\rho (\text{r})=\rho_{0}(\text{r})+\delta \rho (\text{r})$$

DFTB3 is based on the expansion of the DFT total energy at $\rho_{0}$(**r**) up to third order in δρ(**r**). Considering this approximation, the DFTB3 total energy can be expressed as

(2) $$\begin{array}{c} \begin{array}{l} E^{\text{DFTB3}}=E^{\text{H0}}+E^{\gamma }+E^{\Gamma }+E^{\text{rep}}\\ \;=\underset{iab}{\sum }\underset{\mu \in a}{\sum }\underset{\nu \in b}{\sum }n_{i}c_{\mu i}c_{\nu i}H_{\mu \nu }^{0}+\frac{1}{2}\underset{ab}{\sum }\Delta q_{a}\Delta q_{b}\gamma_{ab}\\ \;+\frac{1}{3}\underset{ab}{\sum }\Delta q_{a}^{2}\Delta q_{b}\Gamma_{ab}+\frac{1}{2}\underset{ab}{\sum }V_{ab}^{\text{rep}}\; \end{array} \end{array}$$

The first term $E^{\text{H0}}$ corresponds to the effect of δρ(**r**) to first order and is equivalent to the sum of all orbital eigenvalues. $n_{i}$ gives the number of electrons that occupy molecular orbital $\psi_{i}$, which is constructed from a linear combination of a minimal basis set of confined pseudoatomic orbitals $\phi_{\mu }$, as expressed in the following Equation (3).

(3) $$\psi_{i}=\underset{\mu }{\sum }c_{\mu i}\phi_{\mu }$$

The minimal basis set is determined via numerical DFT calculations of confined spherical atoms employing the following equation

(4) $$\left[\hat{T}+V^{\text{nucleus}}+V^{\text{Hartree}}+V^{\text{xc}}+{\left(\frac{r}{r^{\text{wf}}}\right)}^{\sigma }\right]\phi_{\mu }=\epsilon_{i}\phi_{\mu }$$

with the exchange correlation potential $V^{\text{xc}}$ being typically handled at GGA level of theory, utilizing the Perdew‐Burke‐Ernzerhof (PBE) exchange correlation functional [65]. The last term within the square brackets of Equation (4) applies a power confinement potential to the basis set functions. σ defines the confinement order and $r^{\text{wf}}$ denotes the associated wave function compression radius. In this work, the tuning parameters σ and $r^{\text{wf}}$ have been optimized independently for all valence subshells. Furthermore, the confinement has not been constrained to a quadratic potential, resulting in molecular orbitals of superior quality and a potential improvement in the overall performance of the generated zirconium parameters [66]. The rationale behind the compression of $\phi_{i}$ is to eliminate the influence of long‐range orbital tails on adjacent basis set functions, thereby facilitating an enhanced representation of solid‐state systems [67]. With all pseudoatomic orbitals being determined, the Hamiltonian matrix elements are computed within a two‐center approximation as

(5) $$\begin{array}{c} H_{\mu \nu }^{0}=\left\{\begin{array}{cc} \epsilon_{\mu }^{\text{isolated}}\; & \text{if}\;\mu =\nu \\ \left\langle \phi_{\mu }\left|\hat{T}+V^{\text{KS}}\right|\phi_{\nu }\right\rangle \; & \text{if}\;a\neq b\\ 0\; & \text{if}\;a=b\;\text{and}\;\mu \neq \nu \end{array}\right. \end{array}$$

In this two‐center approximation, all $H_{\mu \nu }^{0}$ depend only on the relative orientation and distance between the basis functions μ and ν, as they are independent of all other basis functions within the system. This allows for the calculation of matrix elements on a discretized grid, which represents the so‐called Slater‐Koster table [68]. The diagonal elements $\epsilon_{\mu }^{\text{isolated}}$ correspond to the eigenvalues of the atomic orbitals in the isolated atom and are obtained by omitting the confinement potential term in Equation (4). The Kohn‐Sham (KS) potential $V^{\text{KS}}$ can be expressed in terms of a potential superposition [61] $\left(V^{\text{KS}}=V^{\text{KS}}(\rho_{0}^{a})+V^{\text{KS}}(\rho_{0}^{b})\right)$ or a density superposition [62] $\left(V^{\text{KS}}=V^{\text{KS}}(\rho_{0}^{a}+\rho_{0}^{b})\right)$. $\rho_{0}^{a}$ denotes the reference electron density of the confined spherical atom a, and it is calculated by replacing $r^{\text{wf}}$ with a compression radius for the electron density $r^{\text{dens}}$. To increase the flexibility of the parametrization, $r^{\text{dens}}$ and the associated confinement order also underwent an optimization procedure. All existing parametrizations within the 3OB set have been carried out using density superposition. However, as outlined by Wahiduzzaman et al. [66], both expansions are formally exact for extension to infinite order.

The second term $E^{\gamma }$ in Equation (2) describes the second‐order contribution of the total energy expansion, with $\Delta q_{a}=q_{a}-q_{a}^{0}$ being the partial charge (negative Mulliken charge) of atom a. The function $\gamma_{ab}$ describes the Coulomb interaction of the net atomic charges of atoms a and b at distance $r_{ab}$ via Equation (6).

(6) $$\gamma_{ab}=\frac{1}{r_{ab}}-S(U_{a},U_{b},r_{ab})\cdot h(U_{a},U_{b},r_{ab})$$

At large interatomic distances, the function becomes equal to the interaction of two infinitesimally small charges. As $r_{ab}$ approaches 0, the short‐ranged function S·h becomes significant, and $\gamma_{ab}$ converges to the atomic Hubbard parameter $U_{a}$, which is determined from an atomic DFT calculation by subtracting the atom's electron affinity from its ionization energy.

The influence of the charge fluctuations to third order is introduced by the third term $E^{\Gamma }$ in Equation (2). The function $\Gamma_{ab}$ is defined as the first derivative of $\gamma_{ab}$ with respect to $q_{a}$, thereby containing the derivative of the atomic Hubbard parameter. To compute the Hubbard derivative $U_{a}^{\text{d}}$, the Hubbard parameter is differentiated with respect to the net atomic charge. By considering the fluctuation terms of second and third order, the energy depends on the partial charges, which in turn depend on the LCAO coefficients $c_{\mu i}$, requiring the equations to be solved self‐consistently. According to the variational ansatz applied to the DFTB3 total energy expression, Equation (2), the Kohn‐Sham equations are written as the following

(7) $$\sum_{b}\sum_{\nu \in b}c_{\nu i}\left(H_{\mu \nu }-\epsilon_{i}S_{\mu \nu }\right)=0\;\forall a,\mu \in a,i$$

with the DFTB3 Hamiltonian matrix elements reflecting the necessity of a self‐consistent solution, as shown below

(8) $$\begin{array}{ll} H_{\mu \nu } & =H_{\mu \nu }^{0}+S_{\mu \nu }\underset{c}{\sum }\Delta q_{c}\left(\frac{1}{2}\left(\gamma_{ac}+\gamma_{bc}\right)+\frac{1}{3}\left(\Delta q_{a}\Gamma_{ac}+\Delta q_{b}\Gamma_{bc}\right)\right.\\ & \;\left.+\frac{1}{6}\;\Delta q_{c}\left(\Gamma_{ca}+\Gamma_{cb}\right)\right)\\ & \;\forall a,b,\mu \in a,\nu \in b\; \end{array}$$

and $S_{\mu \nu }=\langle \phi_{\mu }|\phi_{\nu }\rangle$ denoting the overlap matrix elements.

The last term contributing to the DFTB3 total energy, as defined in Equation (2), represents the repulsive energy $E^{\text{rep}}$ as the sum of pairwise additive repulsive potentials, denoted as $V_{ab}^{\text{rep}}$. These short‐ranged repulsive potentials serve as a catch‐all term to compensate for the approximations made by being tailored to reproduce computationally determined reference data at high level of theory. $V_{ab}^{\text{rep}}$ is commonly represented using polynomials [61, 69], splines [70, 71], or more recently machine learning models [72, 73]. In the spirit of following the original 3OB parametrization, the spline‐based protocol as outlined by Gaus, Goez, and Elstner has been followed in this work [44].

## 3 Parametrization of Zirconium

The process of creating DFTB parameters can be divided into two principal stages. The initial stage involves the optimization of the electronic parameters, which pertain to the first three terms in Equation (2). The eight electronic one‐center parameters to be adjusted are the compression radii and the confinement order for all valence subshells ($r^{\text{s}}$, $r^{\text{p}}$, $r^{\text{d}}$, $\sigma^{\text{s}}$, $\sigma^{\text{p}}$, $\sigma^{\text{d}}$), in addition to those of the electron density ($r^{\text{dens}}$, $\sigma^{\text{dens}}$). It should be noted that the orbital eigenvalues of zirconium $\epsilon_{\text{s}}$, $\epsilon_{\text{p}}$, and $\epsilon_{\text{d}}$, as well as the atomic Hubbard parameter and its derivative have not been optimized in the present work. To ensure consistency with the 3OB parametrization, all calculations of zirconium one‐center parameters and Hamiltonian matrix elements have been carried out at DFT level of theory, employing the PBE functional for exchange and correlation [65]. The Libxc‐6.0.0 library [74] and Hotcent‐2.0.0 [75] have been used to perform the calculations.

In the second stage of the parametrization, Tango‐1.0.0 [76] has been utilized to optimize the repulsive potentials, which constitute the last term of Equation (2). All DFTB calculations in this work have been performed via the DFTB+ program package version 24.1 [77].

### 3.1 Fitting of Electronic Parameters

Firstly, $\epsilon_{\text{s}}$, $\epsilon_{\text{p}}$, $\epsilon_{\text{d}}$, $U_{a}$, and $U_{a}^{\text{d}}$ have been determined for the isolated, unconfined, spin‐unpolarized, spherical zirconium atom through an all‐electron atomic DFT calculation. To optimize the parameters of the confinement potentials, the band structure of hexagonal zirconium in space group no. 194 has been iteratively calculated via DFTB and fitted to a reference band structure using the gradient‐less COBYLA algorithm [78]. The reference band structure has been computed from an energy minimization at DFT level of theory using the PBE exchange correlation functional as implemented in Crystal23 [79] and the pob‐TZVPD‐rev2 basis set [80]. To ascribe greater significance to the electronic bands in proximity to the Fermi level, Boltzmann‐distributed weights, as described in Equation (9), with a value for $k_{\text{B}}T$ of 0.01 eV have been employed.

(9) $$w=\exp\left(-\frac{\left|\epsilon -E_{\text{Fermi}}\right|}{k_{\text{B}}T}\right)$$

**FIGURE 1:**
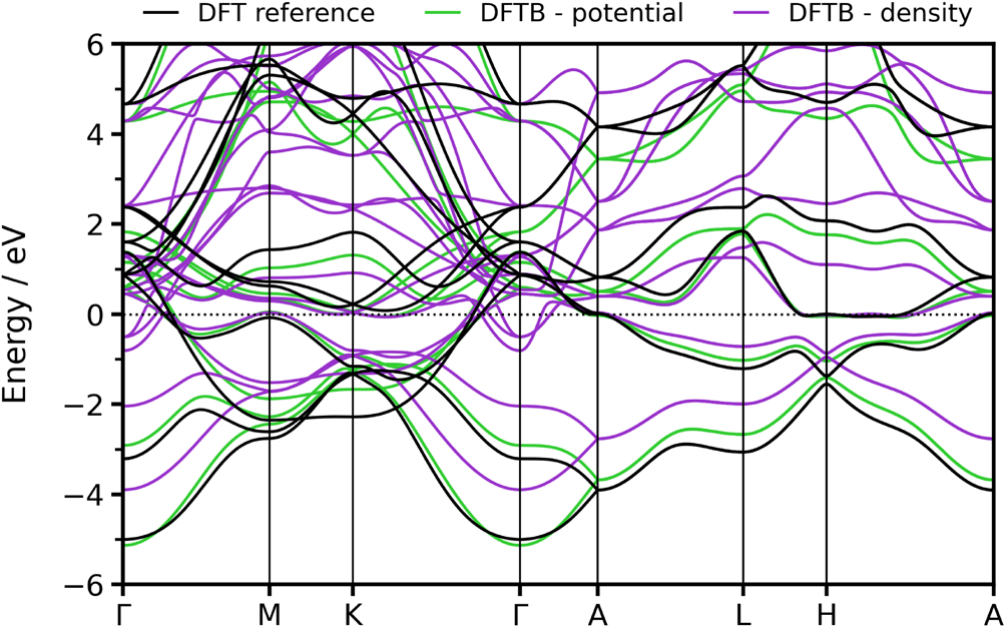
Comparison of the band structure of Zr (hcp) calculated using DFTB in conjunction with the potential (green) and the density superposition scheme (purple) against the band structure computed via DFT at PBE level of theory (black).

To achieve the optimal set of electronic parameters, both the potential and the density superposition scheme have been subjected to testing. A comparison of the resulting band structures with the reference is shown in Figure 1. As previously indicated in the literature [66] and validated in this study, expressing the KS potential via a potential superposition produces superior results when used for the purpose of band structure fitting. Henceforth, the potential superposition scheme has been chosen for the determination of the electronic one‐center parameters of zirconium, which are presented in Table 1, as well as for the calculation of all Slater‐Koster tables involving zirconium. However, the existing 3OB parametrizations have been developed exclusively using a pairwise superposition of atomic electron densities. Since it was unclear whether mixing the two superposition schemes would yield a successful extension of the 3OB set for Zr, a comprehensive benchmark has been conducted. This process included calculating more than 1,800 reference structures sourced from the CSD and performing MD simulations of two particularly challenging Zr‐based MOF systems, in addition to investigating ligand association and exchange reaction energies commonly presented in 3OB parametrizations [44, 58, 59, 60].

**TABLE 1:** Summary of the electronic parameters of zirconium^a^.

Parameter	Value
$l_{\max}$	2
$\epsilon_{\text{s}}$	−0.1631482
$\epsilon_{\text{p}}$	−0.0540024
$\epsilon_{\text{d}}$	−0.1322810
$U_{a}$	0.3866978
$U_{a}^{\text{d}}$	−0.05538
$r^{\text{s}}$	4.90
$r^{\text{p}}$	5.53
$r^{\text{d}}$	4.71
$r^{\text{dens}}$	9.40
$\sigma^{\text{s}}$	1.84
$\sigma^{\text{p}}$	5.53
$\sigma^{\text{d}}$	14.1
$\sigma^{\text{dens}}$	2.59

^a^ Parameters have been calculated using the potential superposition scheme. All values are given in atomic units unless they are unitless.

### 3.2 Fitting of Repulsive Potentials

All 16 repulsive potentials $V_{ab}^{\text{rep}}$ have been tuned to reproduce potential energy curves generated from selected reference structures, which are listed in Table 2. In the case of the Zr‐Zr repulsive potential, the zirconium (hcp) structure optimized in the course of the band structure fitting has been isotropically contracted and expanded from 88% to 125% in increments of 1% to generate the potential energy curve. This has again been carried out via the Crystal23 program at PBE level of theory employing the pob‐TZVPD‐rev2 basis set. For all other elements X, each reference compound has been subjected to an energy minimization prior to scanning the Zr‐X bond of interest. Møller‐Plesset perturbation theory of second order (MP2) [81], as implemented in Gaussian 16 [82], has been selected for this process together with the cc‐pVTZ basis set [83, 84, 85, 86, 87, 88] obtained from the Basis Set Exchange software [89, 90, 91]. Effective core potentials have been employed for zirconium and iodine by utilizing the cc‐pVTZ‐PP basis set [92, 93, 94]. Regarding potassium, no correlation‐consistent polarized non‐relativistic triple‐zeta basis set is currently known to exist. Given its similarity and compatibility with the MP2 scheme, the def2‐TZVPPD basis set was selected for the calculations including K [95, 96]. The repulsive potentials are visualized in Figures S1–S16.

**TABLE 2:** Reference structures used to determine the Zr‐X repulsive potentials.

X	Reference structures
H	[ZrH 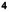 ]
C	[Zr(CH 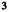 ) 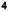 ], [Zr(CH 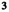 ) 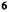 ] 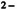 , [ZrH 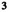 (CH 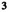 )], [ZrH 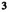 (CN)]
N	[Zr(NH 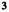 ) 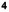 ] 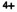 , [Zr(NH 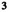 ) 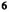 ] 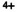
O	[Zr(H 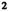 O) 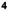 ] 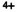 , [Zr(H 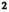 O) 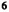 ] 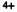 , [Zr(H 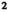 O) 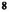 ] 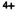 , [Zr(OH) 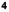 ]
F	[ZrH 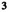 F], [Zr(NH 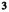 ) 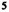 F] 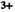
Na	ZrHNa
Mg	ZrMg
P	[Zr(PH 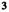 ) 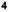 ] 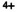 , [Zr(PH 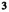 ) 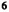 ] 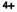
S	[Zr(SH 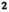 ) 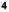 ] 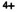 , [Zr(SH 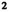 ) 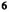 ] 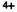 , [Zr(SH) 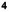 ]
Cl	[ZrH 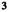 Cl], [Zr(NH 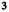 ) 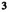 Cl] 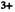 , [Zr(NH 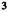 ) 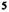 Cl] 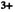
K	ZrHK
Ca	ZrCa
Zn	ZrZn
Br	[Zr(NH 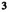 ) 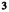 Br] 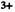 , [Zr(NH 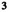 ) 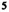 Br] 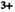
Zr	Zr (hcp)
I	[Zr(NH 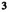 ) 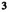 I] 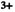 , [Zr(NH 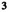 ) 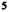 I] 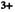

## 4 Benchmarks and Discussion

This section presents the results of the parametrization for Zr in conjunction with the 3OB parameters for C, H, N, O, S, P, Mg, Zn, Na, K, Ca, F, Cl, Br, and I. Comparisons have been drawn with respect to MP2 and PBE, as both methods have been utilized in the course of the parametrization process. Given the popularity of this approach within the chemical‐scientific community and its demonstrated accuracy [97, 98], B3LYP [99] calculations have also been carried out for comparative purposes. Furthermore, recently developed semi‐empirical methods, namely DFTB2/PTBP [100] (PTBP) and GFN2‐xTB [101] (xTB), as well as the machine learning approach MACE‐MP‐0 [102, 103, 104, 105] (MACE), have been considered. For all DFT, DFTB, and MACE calculations, third‐order dispersion correction according to Grimme et al. [106], in conjunction with Becke‐Johnson damping [107], has been employed. In the case of the DFTB calculations, to account for the relatively small covalent radius of the hydrogen atom, all interactions involving H have been corrected via the damping factor $h(U_{a},U_{b},r_{ab})$ in Equation (6), using a value of 4.0 for the ζ parameter [60]. The large MACE‐MP‐0 model has been chosen for all calculations denoted as MACE.

### 4.1 Zirconium‐Ligand Association and Reaction Energies

Small zirconium complexes in the gas phase have been considered in the first part of the benchmark. We define zirconium‐ligand association energies

(10) $$\begin{array}{ll} {\text{ZrL}}_{\text{x}}^{4+}+\text{L} & \to {\text{ZrL}}_{\text{x+1}}^{4+}\; \end{array}$$

(11) $$\begin{array}{ll} {\text{ZrL}}_{\text{x}}^{(\text{x}-4)-}+{\text{L}}^{-} & \to {\text{ZrL}}_{\text{x+1}}^{(\text{x}-3)-}\; \end{array}$$

(12) $$\begin{array}{ll} \text{ZrM}+\text{M} & \to {\text{ZrM}}_{2}\; \end{array}$$

for Zr(IV) complexes with neutral L and negatively charged ligands ${\text{L}}^{-}$ and Zr(0) with neutral metal atoms M. The results yielded by the newly developed parameters, as well as those obtained via the two DFT functionals PBE and B3LYP, and the two DFTB approaches PTBP and xTB, have been contrasted with those obtained through MP2, as it is known to provide accurate energy estimates at reasonable computational cost [108, 109, 110]. For MP2 and B3LYP calculations, the cc‐pVTZ basis set [83, 84, 85, 86, 87, 88] with effective core potentials for Zr and I [92, 93, 94] and the def2‐TZVPPD basis set [95, 96] for K have been employed. For DFT calculations with the PBE functional, the def2‐SVP basis set [92, 95, 111] has been selected. The findings of these comparisons are presented in Table 3. To facilitate a direct comparison of the potential energy surfaces at varying levels of theory, zero‐point energies (ZPEs) have not been included. This strategy is consistent with the 3OB parametrization protocols that have been previously established [44, 53, 58, 59]. The energy values obtained from the 3OB extension demonstrate strong agreement with those yielded from MP2 calculations, exhibiting a mean absolute deviation (MAD) of 3.5 kcal/mol. This observed magnitude of deviation is comparable to those reported in the parametrization of other metals, such as magnesium, zinc, and copper [53, 59]. PBE exhibits the largest MAD of 21.8 kcal/mol with particular difficulties in the description of negatively charged ligand and metal association energies. Conversely, B3LYP demonstrates a strong match with MP2 for neutral ligands and good agreement for negatively charged ones. However, a notable exception is observed for the ZrZn compound, with a deviation of 20.6 kcal/mol. PTBP and xTB exhibit comparable MAD values of 13.2 kcal/mol and 15.1 kcal/mol, as well as maximum absolute deviation (MAX) values of about 50 kcal/mol.

**TABLE 3:** Zirconium‐ligand association energies relative to MP2 reference values^a^.

Complex^b^	MP2^c^	3OB	PBE^d^	B3LYP^c^	PTBP	xTB
[Zr(H 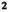 O) 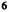 ] 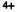	−57.9	+1.6	−6.4	−0.6	+3.8	−17.5
[Zr(H 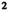 O) 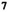 ] 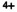	−53.3	+5.9	−5.9	0.0	+4.8	−15.9
[Zr(H 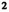 O) 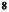 ] 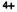	−34.8	+3.3	−6.1	+0.1	−3.2	−19.6
[Zr(NH 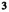 ) 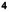 ] 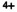	−93.0	+1.1	−6.5	−0.9	+26.0	−10.1
[Zr(NH 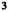 ) 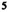 ] 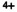	−79.7	+4.5	−5.5	−0.1	+19.4	−0.3
[Zr(SH 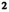 ) 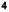 ] 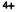	−71.0	+2.2	−5.2	−0.4	+8.2	+5.5
[Zr(SH 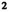 ) 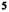 ] 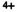	−60.5	+1.5	−4.9	−0.1	+5.2	+16.1
[Zr(PH 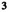 ) 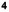 ] 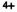	−74.7	−0.5	−4.0	0.0	+3.3	−2.5
[Zr(PH 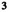 ) 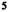 ] 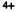	−65.3	+6.4	−2.2	+0.7	−2.7	+7.5
ZrMg	−7.8	−2.6	−6.9	0.0	−50.2	−27.0
ZrCa	−5.6	+2.5	−33.6	−20.6	−12.2	−51.3
ZrZn	+0.1	−4.2	−16.0	−7.7	−33.7	+39.3
[ZrH 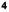 ]	−88.0	−13.4	−6.4	+5.9	−1.7	−5.3
[Zr(CH 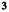 ) 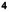 ]	−82.7	+8.2	+0.4	+5.2	−25.3	+2.9
[ZrH 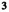 F]^e^	−121.3	+1.0	−29.3	+1.1	+5.7	−2.7
[ZrH 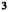 Cl]^e^	−73.4	+0.2	−22.0	+2.5	−9.2	−5.8
[ZrH 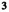 Br]^e^	−65.1	+0.7	+228.1	+3.9	−8.5	−15.9
[ZrH 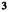 I]^e^	−58.4	+3.5	−2.7	+2.9	−15.3	−26.8
MAD		3.5	21.8	2.9	13.2	15.1
MAX		13.4	228.1	20.6	50.2	51.3

^a^ Energies are given in kcal/mol and have been computed at 0 K, excluding ZPE.

^b^ The complex before ligand association is shown.

^c^ Basis set cc‐pVTZ with effective core potentials for Zr and I.

^d^ Basis set def2‐SVP.

^e^ An additional halogen of the same element is being associated.

The same methodologies have been employed in the study of various zirconium complex ligand exchange reaction energies, which are summarized in Table 4. The parameters presented in this work achieve an excellent agreement with energies obtained through MP2 calculations with a mean absolute deviation as low as 3.8 kcal/mol. Once more, PBE displays the most substantial overall energy deviation, exhibiting a MAD of 68.7 kcal/mol. In general, PBE encounters considerable challenges in the accurate energetic description of the investigated bromine‐containing complexes. B3LYP exhibits slightly reduced accuracy compared to the calculated association energies but provides analogous values to those yielded by PTBP and xTB. The latter demonstrates significant improvements in comparison to its description of ligand association energies. These results for the zirconium‐ligand association and reaction energies support the application of the potential superposition scheme for determining the electronic parameters of Zr and the subsequent mixing of potential and density superposition within this extension of the 3OB parameter set.

**TABLE 4:** Zirconium‐ligand exchange reaction energies relative to MP2 reference values^a^.

Reaction	MP2^b^	3OB	PBE^c^	B3LYP^b^	PTBP	xTB
K + ZrHNa → Na + ZrHK	−1.8	+4.9	−4.9	−1.4	+5.9	+11.5
Ca + ZrMg → Mg + ZrCa	+4.0	−3.5	−11.9	−9.3	+11.6	+7.1
Cl 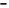 + [ZrH 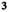 F] → F 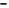 + [ZrH 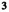 Cl]	+50.9	−4.8	+17.2	+11.7	−5.0	+7.0
I 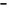 + [Zr(NH 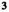 ) 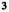 Br] 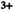 → Br 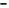 + [Zr(NH 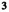 ) 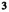 I] 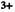	+11.1	+2.1	−241.1	−13.1	−12.6	−2.0
MAD		3.8	68.7	8.9	8.8	6.9
MAX		4.9	241.1	13.1	12.6	11.5

^a^ Energies are given in kcal/mol and have been computed at 0 K, excluding ZPE.

^b^ Basis set cc‐pVTZ with effective core potentials for Zr and I and basis set def2‐TZVPPD for K.

^c^ Basis set def2‐SVP.

### 4.2 Root Mean Square Deviation

**FIGURE 2:**
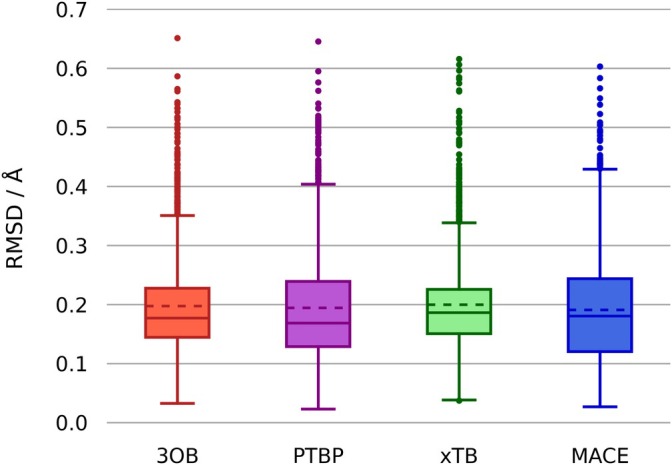
Root mean square deviation of 1,897 zirconium‐containing structures optimized at DFTB3/3OB, DFTB2/PTBP, GFN2‐xTB, and MACE‐MP‐0 level of theory relative to the original CSD structures. Dashed lines indicate the mean value of each data set and solid lines the median. Whiskers extend to the farthest point within 1.5 times the interquartile range. Outliers are marked as solid dots, with the numbers for the individual methods being 3OB: 108, PTBP: 67, xTB: 125, and MACE: 37.

**FIGURE 3:**
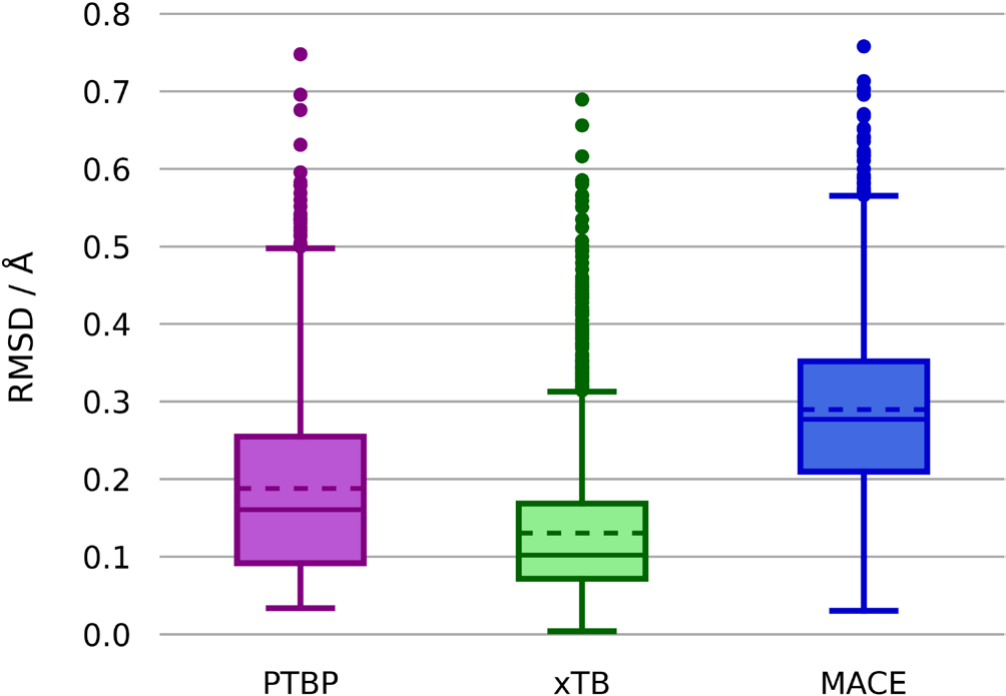
Root mean square deviation between the structures optimized at DFTB3/3OB level of theory and the three reference approaches DFTB2/PTBP, GFN2‐xTB, and MACE‐MP‐0. A total of 1,897 zirconium‐containing structures have been taken from the CSD. Dashed lines mark the mean value of each data set and solid lines the median. Whiskers extend to the farthest point within 1.5 times the interquartile range. Outliers are marked as solid dots, with the numbers for the individual methods being: PTBP: 27, xTB: 96, and MACE: 34.

To validate the ability to reproduce a wide variety of geometries, a search of the Cambridge Structural Database has been conducted using the CSD Python API (v3.3.0) [112, 113] for zirconium‐containing compounds. The 1,897 filtered structures have been subjected to energy minimizations via the Atomic Simulation Environment (ASE) framework (v3.23.0) [114] utilizing the LBFGS [115] optimizer at DFTB3/3OB, DFTB2/PTBP, GFN2‐xTB and MACE‐MP‐0 level of theory, respectively. A corresponding spreadsheet containing detailed information on all used structures is provided in the Supporting Information. To assess the quality of the newly developed parameters, the root mean square deviation (RMSD) based on the Kabsch algorithm [116] has been calculated in relation to the structures optimized by the various methods, as well as relative to the configurations reported in the CSD. Due to the rotational invariance of methyl and amine groups, hydrogen atoms have been excluded in the calculation of the RMSD values. Additionally, unreasonable initial configurations have been sorted out by neglecting structures in which any atom has moved more than 1 Å in the course of the optimization. The results have been visualized in Figures 2 and 3. As illustrated by the comparison to the initial structures deposited in the CSD (Figure 2), the novel 3OB extension demonstrated comparable performance to the other tested semi‐empirical approaches. Of the 1,897 structures examined, 3OB, PTBP, xTB, and MACE exhibit a total of 108, 67, 125, and 37 outliers, respectively. Moreover, the direct comparison of other methodologies to the 3OB parameters (Figure 3) reveals that xTB is the most similar approach with regard to this particular benchmark set. In this comparison, PTBP, xTB, and MACE exhibit 27, 96, and 34 outliers, respectively. The observation that PTBP and MACE have a smaller number of outliers in both cases can be attributed to their larger interquartile range. A histogram illustrating the mass distribution of all utilized CSD structures, along with a comprehensive list of all outliers, can be found in Figure S17 and Tables S1–S7, respectively. The previously described mixing of superposition schemes within this extension of the 3OB parameter set is further substantiated through the presented RMSD values.

### 4.3 Zirconium Desferrioxamine B

**FIGURE 4:**
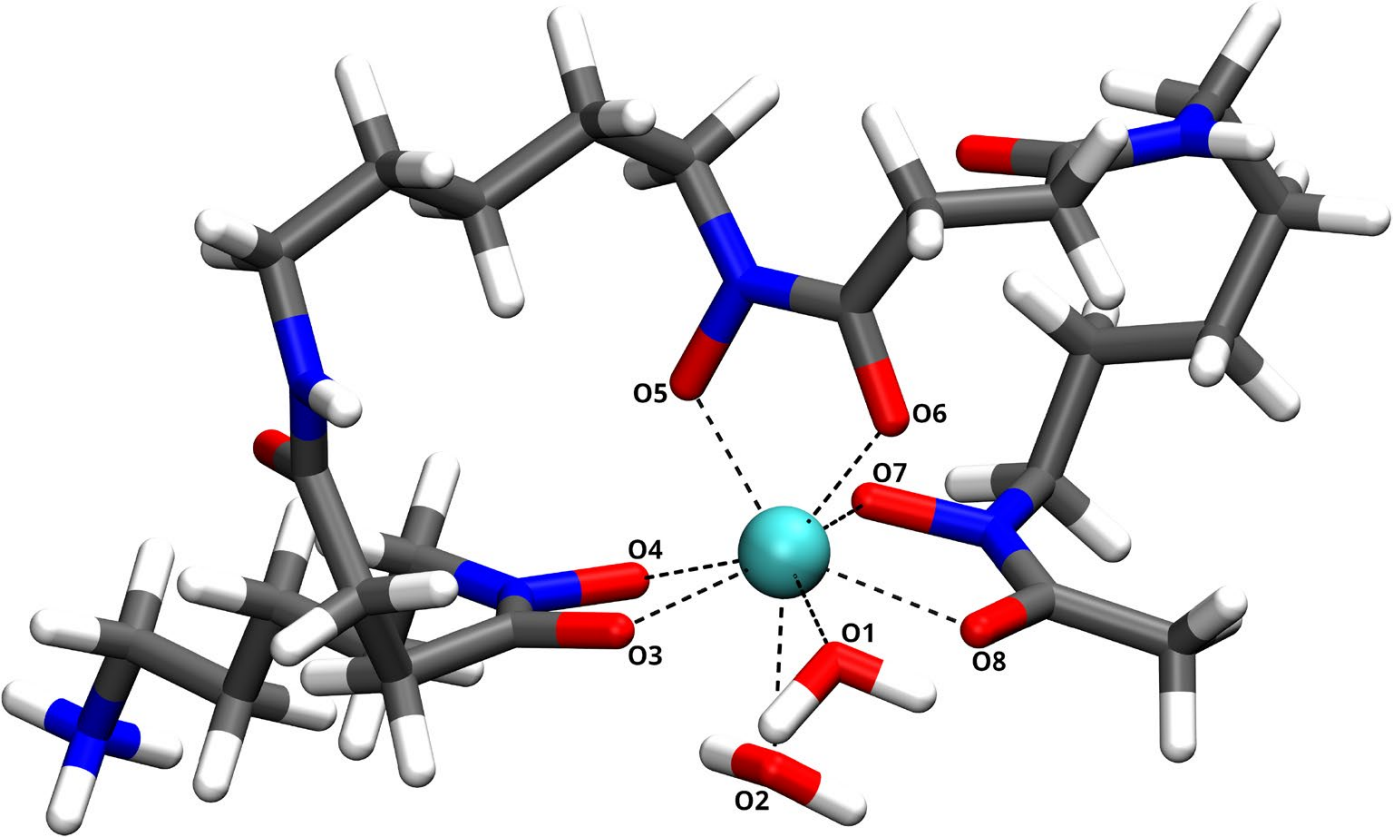
Structure of Zr‐DFO (3‐*cis*) with coordinating oxygen atoms labeled as presented in Table 5. Color code: turquoise: Zr; grey: C; red: O; blue: N; white: H.

To illustrate the applicability of the new 3OB extension to biomedically relevant compounds, a structural investigation of the complex zirconium desferrioxamine B has been conducted. The initial structure of Zr‐DFO has been adopted from the publication by Holland et al. [57] in its most stable 3‐*cis* form, visualized in Figure 4. This configuration has been obtained through energy minimization at DFT level of theory utilizing the B3LYP hybrid functional. In their study, Holland et al. employed the LANL2DZ basis set [117] including an effective core potential for zirconium and the 6‐31+G(d) basis set [118, 119, 120, 121] for the elements H, C, N and O. To facilitate a more robust comparison between the DFT and DFTB approaches, this structure of Zr‐DFO (3‐*cis*) has undergone a re‐equilibration under identical conditions, augmented by the third‐order dispersion correction according to Grimme et al. [106], in conjunction with Becke‐Johnson damping [107]. A subsequent energy minimization via DFTB3/3OB reveals an RMSD of 0.299 Å relative to the B3LYP optimized structure including dispersion correction and damping. As in the CSD calculations, the hydrogen atoms have been excluded from the computation of the RMSD to account for the rotational invariance of methyl and ammonium groups. A detailed comparison of the zirconium‐oxygen distances at the central metal site is listed in Table 5. The comparison yields a mean absolute deviation of 0.118 Å, with the majority of distances demonstrating excellent agreement with the DFT reference calculation. A notable difference is observed for the Zr‐O4 interaction, where DFTB predicts a distance 0.377 Å longer than the DFT optimization. This finding, however, cannot be attributed exclusively to the pure Zr‐O interaction, as the hexadentate DFO ligand is sixfold coordinating the Zr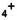 cation. Torsions in the backbone inevitably affect the geometry of the central metal site. Conversely, the two independently coordinating oxygen atoms of the water ligands show deviations as small as 0.003 Å (0.1%) and 0.062 Å (2.7%).

**TABLE 5:** Comparison of Zr‐O distances at the central metal site of Zr‐DFO^a^.

Interaction	DFT/B3LYP^b^	DFTB/3OB
Zr‐O1	2.329	2.267
Zr‐O2	2.453	2.450
Zr‐O3	2.246	2.128
Zr‐O4	2.205	2.582
Zr‐O5	2.107	2.025
Zr‐O6	2.191	2.021
Zr‐O7	2.115	2.074
Zr‐O8	2.257	2.345

^a^ Distances are given in Å.

^b^ Basis set LANL2DZ for Zr and 6‐31+G(d) for H, C, N, and O.

### 4.4 MD Simulation of UiO‐66 and UiO‐67

To assess the precision with which zirconium‐containing MOFs can be represented, UiO‐66 and UiO‐67 have been subjected to MD simulations at DFTB3/3OB, DFTB2/PTBP, and MACE‐MP‐0 level of theory and compared to structures found in the literature. The initial structures of the two MOFs underwent an energy minimization utilizing the novel parameters via the DFTB+ software. The PQ‐0.4.5 software [122] has been used to carry out the simulations, employing the velocity Verlet integrator [123] for time integration. The temperature has been set to 298 K using the Bussi‐Donadio‐Parrinello thermostat [124] with a relaxation time of 0.1 ps and pressure coupling has been enabled by applying the Berendsen manostat [125] with a target pressure of 1.013 bar and a relaxation time of 10 ps. All structures have been placed in a cubic unit cell and have been treated under isotropic conditions. After initial equilibration under NVT and then NPT conditions, both MOFs have been sampled for a total of 200 ps at all three levels of theory, respectively. The resulting time‐averaged lattice parameters are listed alongside reported literature values in Tables 6 and 7. Regarding both MOFs, the time‐averaged lattice parameters calculated from 3OB MD simulations of 20.941Å and 27.050 Å show, in consistency with other theoretical methodologies, a slight overestimate compared to experimental approaches. Notably, the observed results align with the reported literature values, thereby reinforcing the reliability and validity of the newly developed parameters.

**TABLE 6:** Comparison of the UiO‐66 lattice parameter a.

Method	a / Å	T / K	Source
XRD	20.62	523	Vornholt et al. [126]
XRPD	20.7004 (2)	298	Lillerud et al. [18]
XRD	20.7465 (2)	100	Øien et al. [127]
MACE MD	20.862	298	This work
PBE OPT	20.866	0	Wu et al. [128]
PTBP MD	20.870	298	This work
3OB MD	20.941	298	This work
PBE OPT	20.96	0	Yang et al. [129]
B3LYP OPT	21.0171	0	Valenzano et al. [130]
MM MD	21.117 (1)	300	Rogge et al. [131]

**TABLE 7:** Comparison of the UiO‐67 lattice parameter a.

Method	a / Å	T / K	Source
XRD	26.8180 (2)	480	Goodenough et al. [132]
XRD	26.85534 (17)	90	Goodenough et al. [132]
MACE MD	26.877	298	This work
XRD	26.8809 (3)	100	Øien et al. [127]
PTBP MD	26.940	298	This work
3OB MD	27.050	298	This work
PBE OPT	27.14	0	Yang et al. [129]
MM MD	27.328 (2)	300	Rogge et al. [131]

**FIGURE 5:**
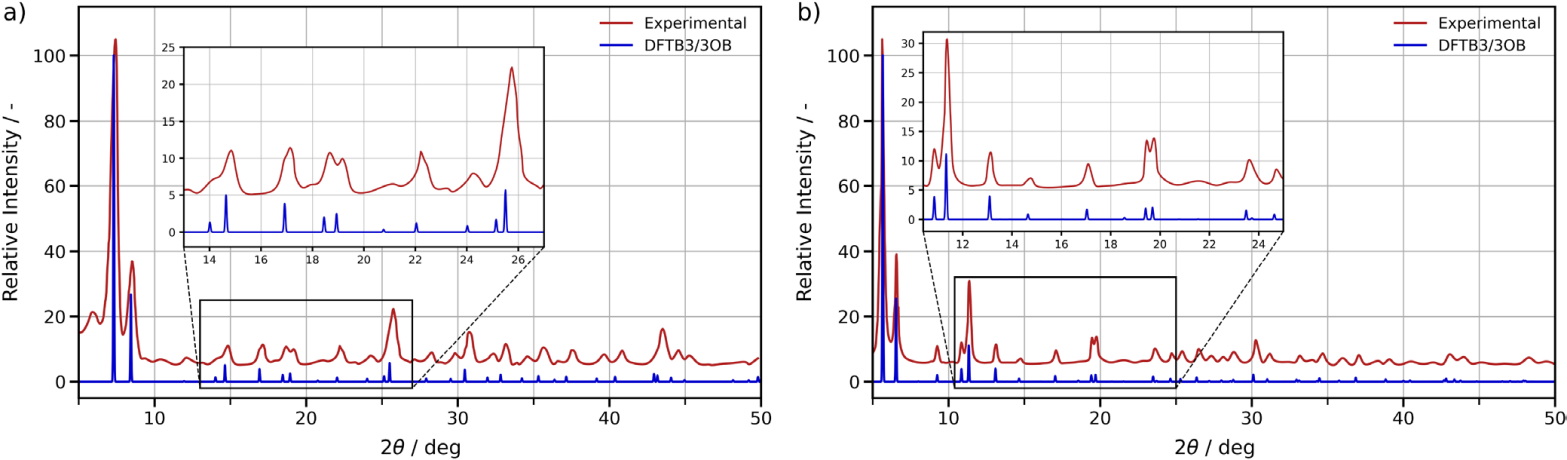
Comparison of calculated X‐ray diffraction patterns (blue) to experimental measurements (red) of a) UiO‐66 and b) UiO‐67. The calculated patterns have been obtained from DFTB3/3OB MD simulations. A wavelength of 1.54060 Å, corresponding to Cu‐K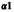 radiation, has been used to compute the theoretical patterns [133, 134].

In addition to the lattice parameters of UiO‐66 and UiO‐67, the ensemble‐averaged X‐ray diffraction patterns have been calculated from the MD simulations and compared to experimental measurements in Figure 5. As illustrated, each theoretically predicted reflex aligns with the experimental reference patterns, further underlining the accuracy of the novel 3OB augmentation.

**FIGURE 6:**
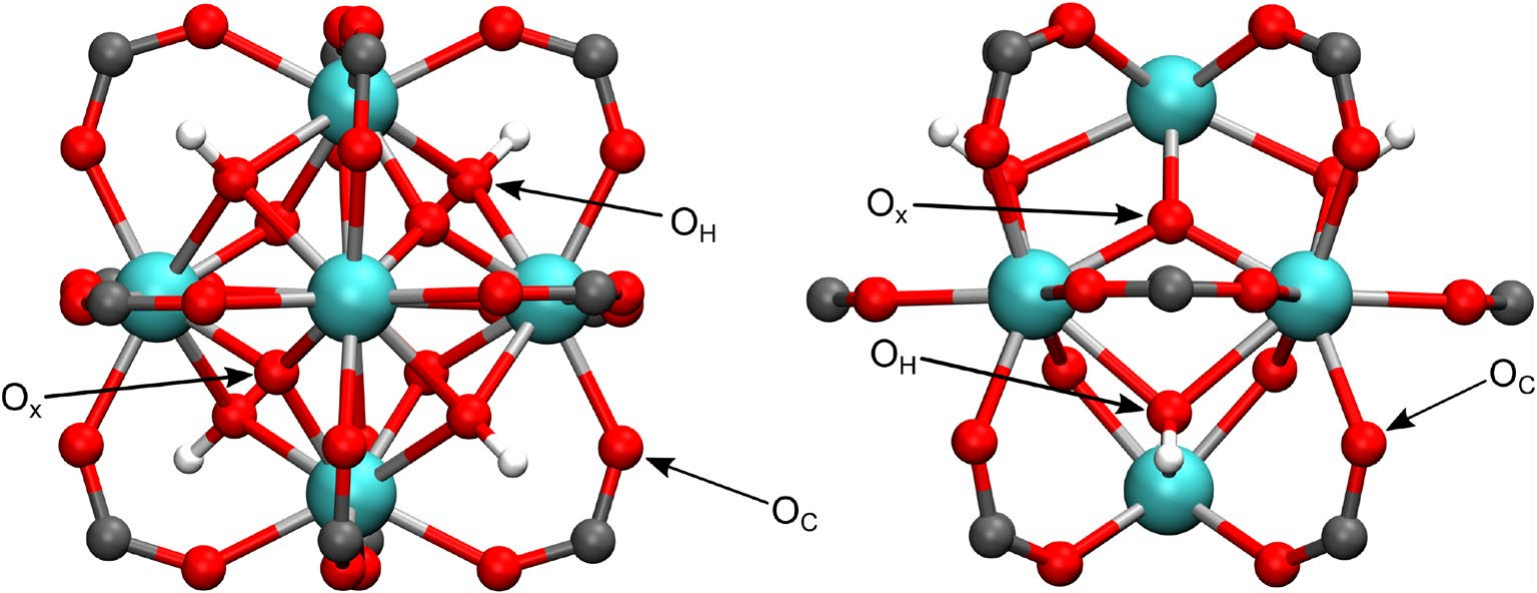
Secondary building unit of the metal‐organic frameworks UiO‐66 and UiO‐67, including the carboxylate groups of the linker molecules. The nomenclature of oxygen atoms used in Tables 8 and 9 is presented from two different viewing directions. Color code: turquoise: Zr; grey: C; red: O; white: H.

Ultimately, interatomic distances within the secondary building unit (SBU) have been evaluated and compared to existing literature, see Tables 8 and 9. The nomenclature of the individual oxygen atom sites has been illustrated in Figure 6. The distance between zirconium and oxide oxygen atoms O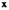 has been found to be in good agreement with the experimental XRD data provided by Øien et al., with a deviation of 0.013 Å (0.6%) for UiO‐66 and 0.006 Å (0.3%) for UiO‐67. For the bond between Zr and hydroxide oxygen atoms O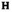, the 3OB extension yields a deviation of 0.024 Å (1.0%) for UiO‐66 and 0.030 Å (1.3%) for UiO‐67 compared to the XRD measurement. An analogous comparison of the distance between zirconium and carboxylate oxygen atoms O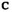 reveals a deviation of 0.027 Å (1.2%) for UiO‐66 and 0.020 Å (0.9%) for UiO‐67. Finally, the Zr‐Zr distance is in excellent agreement with the experimental data, with a deviation of 0.003 Å (0.1%) for UiO‐66 and 0.004 Å (0.1%) for UiO‐67. Overall, the novel parameters provide a more accurate representation of the SBUs than all other tested computational approaches.

**TABLE 8:** Comparison of selected internal coordinates of the secondary building unit of UiO‐66 in Å.

Method	Zr‐O 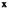	Zr‐O 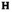	Zr‐O 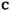	Zr‐Zr	T / K	Source
3OB MD	2.0526	2.2833	2.2374	3.5156	298	This work
XRD	2.065 (3)	2.259 (7)	2.210 (5)	3.513	100	Øien et al. [127]
MM MD	2.086 (3)	2.269 (2)	2.3140 (4)	3.509 (2)	300	Rogge et al. [131]
B3LYP OPT	2.089	2.286	2.249	3.571	0	Valenzano et al. [130]
XRPD	2.118		2.266	3.531	N/A	Valenzano et al. [130]
EXAFS	2.087	2.235		3.511	N/A	Valenzano et al. [130]
MACE MD	2.0964	2.2731	2.2521	3.5555	298	This work
PTBP MD	2.1151	2.2658	2.2338	3.5574	298	This work

**TABLE 9:** Comparison of selected internal coordinates of the secondary building unit of UiO‐67 in Å.

Method	Zr‐O 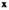	Zr‐O 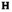	Zr‐O 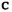	Zr‐Zr	T / K	Source
3OB MD	2.0529	2.2842	2.2385	3.5162	298	This work
XRD	2.059 (2)	2.254 (5)	2.218 (1)	3.512	100	Øien et al. [127]
MM MD	2.085 (3)	2.271 (3)	2.318 (3)	3.513 (3)	300	Rogge et al. [131]
MACE MD	2.0966	2.2733	2.2524	3.5553	298	This work
PTBP MD	2.1150	2.2658	2.2343	3.5577	298	This work

## 5 Conclusion

Zirconium is a key element in the domain of thermo‐chemical stable MOF materials as well as in biomedically relevant drug molecules. The development of computational approaches that are both accurate and expeditious is essential for elucidating microscopical, as well as macroscopical, properties of these structures. In this work, an extension of third‐order density functional tight binding, DFTB3, to zirconium has been presented. The parametrization has been carried out in accordance with previous extensions of the DFTB3/3OB set, which has been shown to provide accurate results in the regime of biomolecules and metal‐organic frameworks. However, a key difference introduced in this work is the mixing of potential superposition with the density superposition scheme, which has been used throughout the 3OB set. The motivation for this choice lies in the improved electronic parameters for zirconium, as evidenced by the enhanced accuracy in calculating the electronic band structure of hexagonal Zr. The validity of this approach has been confirmed through extensive benchmarking.

Calculations of small zirconium‐containing complexes lead to the conclusion that the newly developed parameters are capable of predicting energetics, with a mean absolute deviation of about 3 to 4 kcal/mol. Complexes have been chosen to represent typical zirconium coordination spheres. Overall, DFTB3/3OB displays an accuracy comparable to B3LYP when estimating zirconium‐ligand association and reaction energies, while outperforming other semi‐empirical methods such as DFTB2/PTBP and GFN2‐xTB. Structures taken from the Cambridge Structural Database have been predicted with accuracy similar to that of DFTB2/PTBP, GFN2‐xTB, and MACE‐MP‐0, as demonstrated by the root mean square deviation of 1,897 compounds. The structural characteristics of the metal coordination site of the immunoPET reagent Zr‐DFO have been delineated with a good degree of precision in comparison to B3LYP reference data. Furthermore, the novel parametrization has been applied to MD studies of UiO‐66 and UiO‐67, two prototypical zirconium‐based MOFs, and has been shown to provide accurate lattice parameters, X‐ray diffraction patterns, and internal coordinates of the secondary building unit. A comparison of the results with experimental data and other computational approaches has been undertaken to demonstrate the reliability and validity of the parameters. Excellent agreement has been observed in the prediction of the interatomic distances within the secondary building units of UiO‐66 and UiO‐67, with a maximum deviation of less than 0.03 Å (1.3%) compared to experimental X‐ray diffraction data.

As was the case with the 3OB extension to Na, K, Ca, F, Cl, Br, and I [60], due to the selection of reference structures for tuning the repulsive potentials, the newly developed parameters are not suited for the description of ionic solids, such as zirconia or zirconium carbide (see Section S1). The herein presented extension of the DFTB3 method is expected to facilitate the in‐depth study of zirconium‐containing structures, including medicinally relevant complexes and metal‐organic framework materials.

## Conflicts of Interest

The authors declare no conflicts of interest.

## Supporting information


**Supplemantary Data S1** graphical representation of the repulsive potentials; visualization of the mass distribution of the used CSD structures; details about the outliers in the RMSD calculations; energy minimization results of zirconia and zirconium carbide; spreadsheet with information about all used CSD structures available; Zr‐3OB sk‐files available.

**Data Files S1**.

## Data Availability

The data that supports the findings of this study are available in the Supporting Information of this article.
