# Supplementary material for: Parametrization of Zirconium for DFTB3/3OB: A Pathway to Study Complex Zr‐Compounds for Biomedical and Material Science Applications
Source: J Comput Chem. 2025 May 26;46(14):e70140. doi: 10.1002/jcc.70140 (PMC12105804; doi:10.1002/jcc.70140)
Supplement: Supplementary file 1 — Supplemantary Data S1 graphical representation of the repulsive potentials; visualization of the mass distribution of the used CSD structures; details about the outliers in the RMSD calculations; energy minimization results of zirconia and zirconium carbide; spreadsheet with information about all used CSD structures available; Zr‐3OB sk‐files available. [file JCC-46-0-s002.pdf]

Supporting Information:

Parametrization of Zirconium for DFTB3/3OB: A Pathway to Study Complex Zr-Compounds for Biomedical and Material Science Applications

Armin Penz, Jakob Gamper, Josef M. Gallmetzer, Felix R. S. Purtscher, and Thomas S. Hofer*

Department of General, Inorganic and Theoretical Chemistry, University of Innsbruck

E-mail: T.Hofer@uibk.ac.at

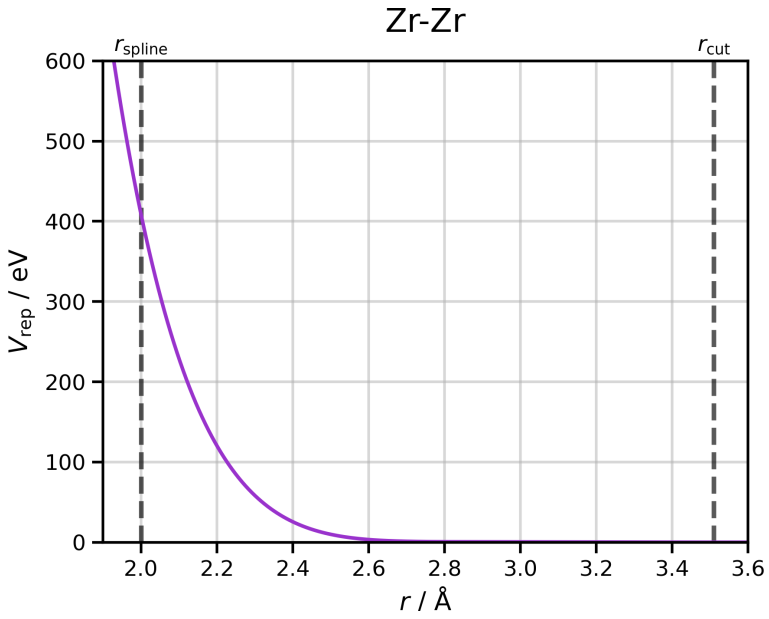

Figure S1: Optimized repulsive potential V_{rep} for the zirconium-zirconium interaction. Following the original 3OB parametrization strategy, the potential is described *via* an exponential function for distances below r_{spline}. In the range between r_{spline} and r_{cut}, the curve is defined by the spline-based protocol. For interatomic distances greater than r_{cut}, V_{rep} is set to zero.

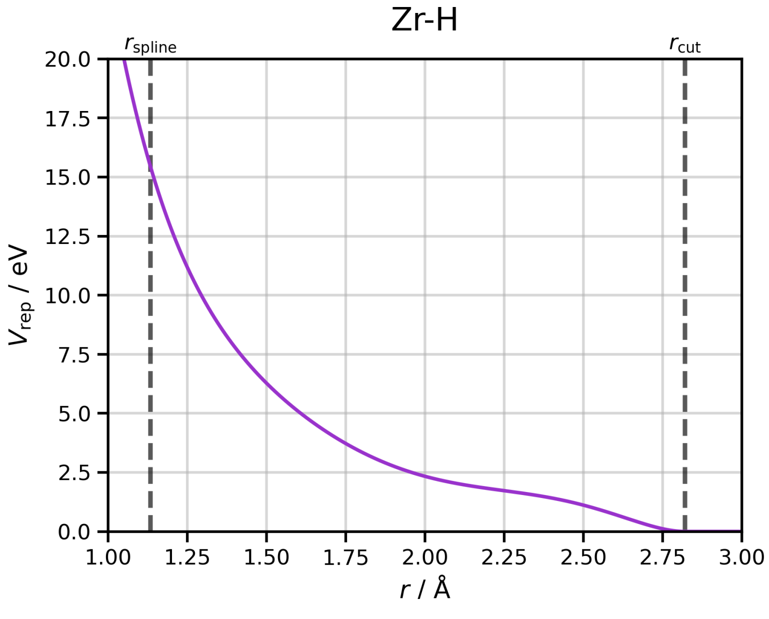

Figure S2: Optimized repulsive potential V_{rep} for the zirconium-hydrogen interaction. Following the original 3OB parametrization strategy, the potential is described *via* an exponential function for distances below r_{spline}. In the range between r_{spline} and r_{cut}, the curve is defined by the spline-based protocol. For interatomic distances greater than r_{cut}, V_{rep} is set to zero.

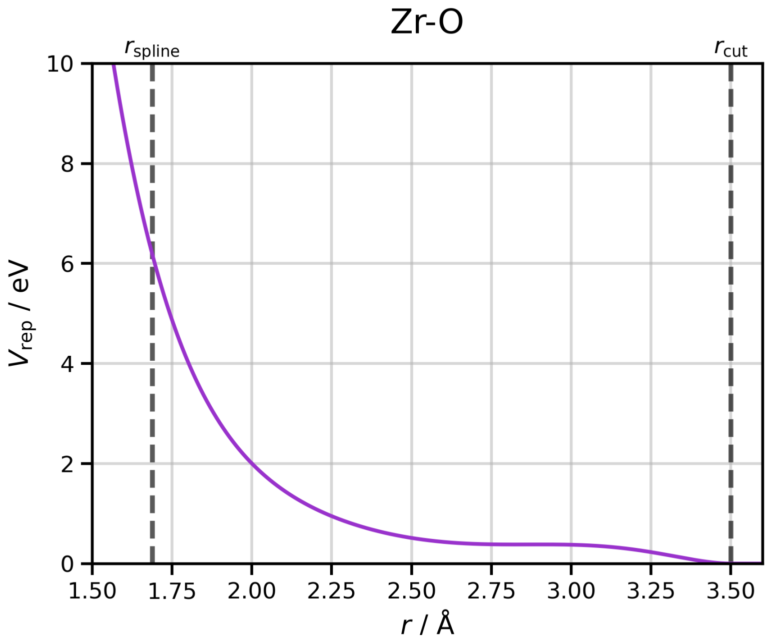

Figure S3: Optimized repulsive potential V_{rep} for the zirconium-oxygen interaction. Following the original 3OB parametrization strategy, the potential is described *via* an exponential function for distances below r_{spline}. In the range between r_{spline} and r_{cut}, the curve is defined by the spline-based protocol. For interatomic distances greater than r_{cut}, V_{rep} is set to zero.

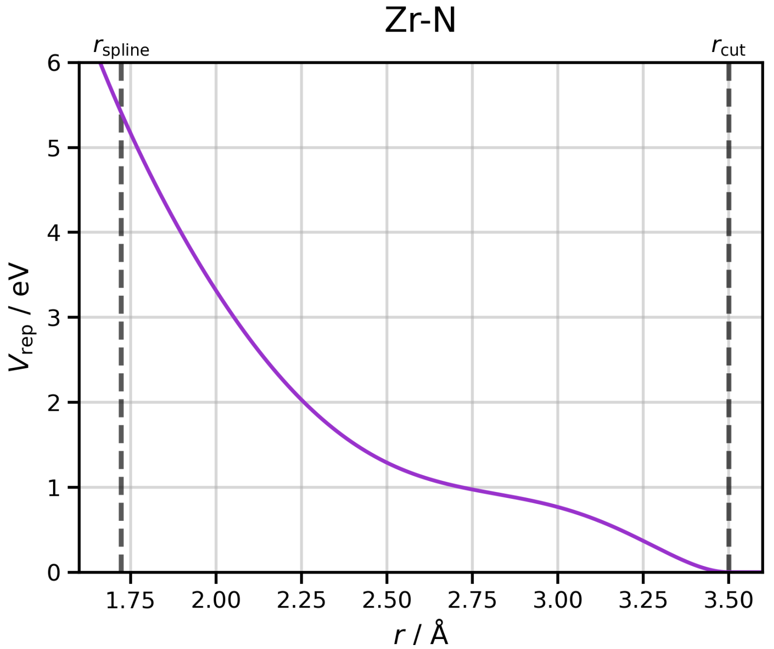

Figure S4: Optimized repulsive potential V_{rep} for the zirconium-nitrogen interaction. Following the original 3OB parametrization strategy, the potential is described *via* an exponential function for distances below r_{spline}. In the range between r_{spline} and r_{cut}, the curve is defined by the spline-based protocol. For interatomic distances greater than r_{cut}, V_{rep} is set to zero.

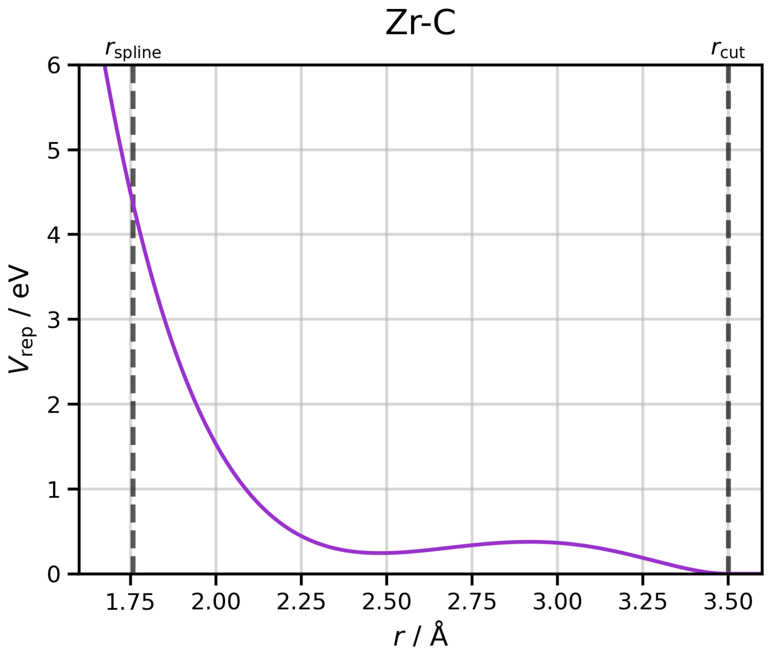

Figure S5: Optimized repulsive potential V_{rep} for the zirconium-carbon interaction. Following the original 3OB parametrization strategy, the potential is described *via* an exponential function for distances below r_{spline}. In the range between r_{spline} and r_{cut}, the curve is defined by the spline-based protocol. For interatomic distances greater than r_{cut}, V_{rep} is set to zero.

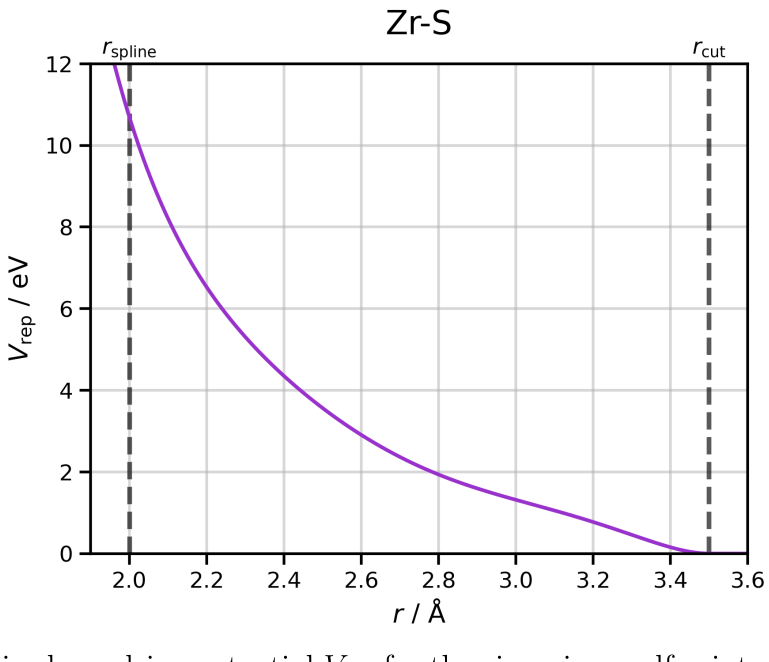

Figure S6: Optimized repulsive potential V_{rep} for the zirconium-sulfur interaction. Following the original 3OB parametrization strategy, the potential is described *via* an exponential function for distances below r_{spline}. In the range between r_{spline} and r_{cut}, the curve is defined by the spline-based protocol. For interatomic distances greater than r_{cut}, V_{rep} is set to zero.

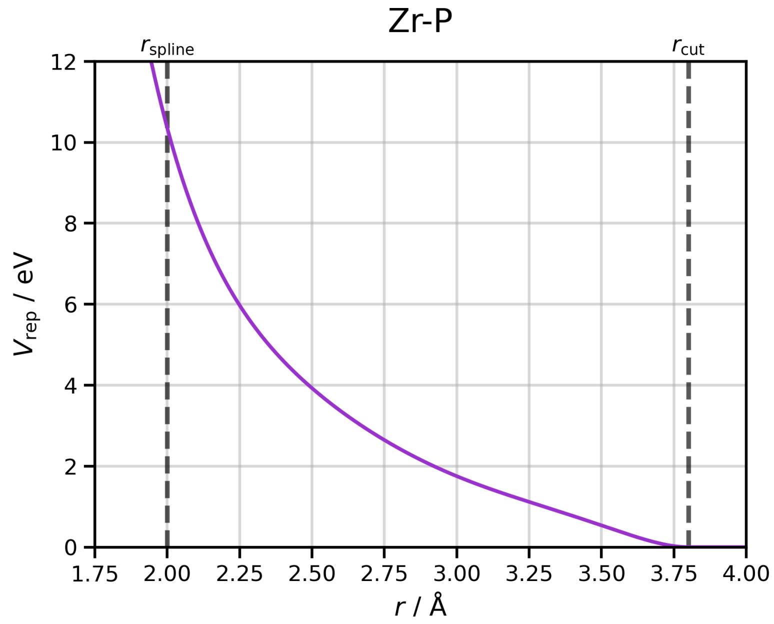

Figure S7: Optimized repulsive potential V_{rep} for the zirconium-phosphorus interaction. Following the original 3OB parametrization strategy, the potential is described *via* an exponential function for distances below r_{spline}. In the range between r_{spline} and r_{cut}, the curve is defined by the spline-based protocol. For interatomic distances greater than r_{cut}, V_{rep} is set to zero.

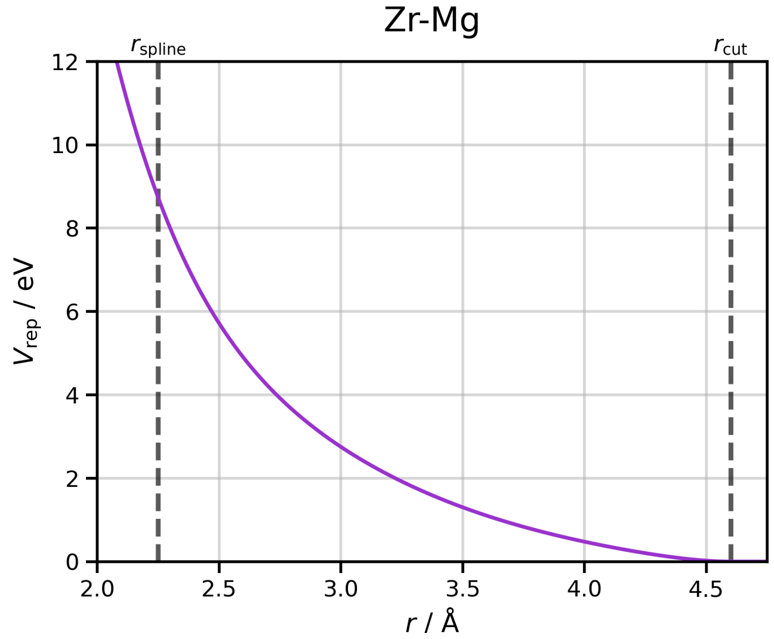

Figure S8: Optimized repulsive potential V_{rep} for the zirconium-magnesium interaction. Following the original 3OB parametrization strategy, the potential is described *via* an exponential function for distances below r_{spline}. In the range between r_{spline} and r_{cut}, the curve is defined by the spline-based protocol. For interatomic distances greater than r_{cut}, V_{rep} is set to zero.

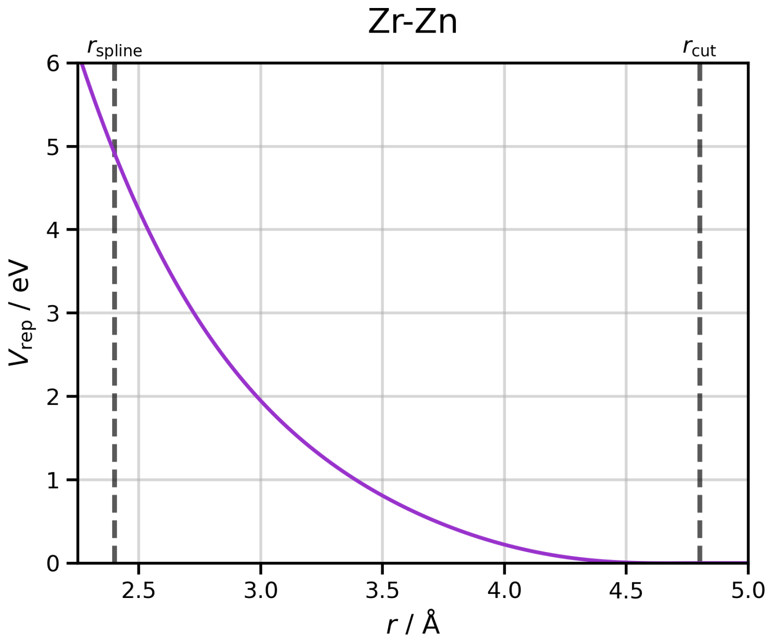

Figure S9: Optimized repulsive potential $V_{\rm rep}$ for the zirconium-zinc interaction. Following the original 3OB parametrization strategy, the potential is described *via* an exponential function for distances below $r_{\rm spline}$. In the range between $r_{\rm spline}$ and $r_{\rm cut}$, the curve is defined by the spline-based protocol. For interatomic distances greater than $r_{\rm cut}$, $V_{\rm rep}$ is set to zero.

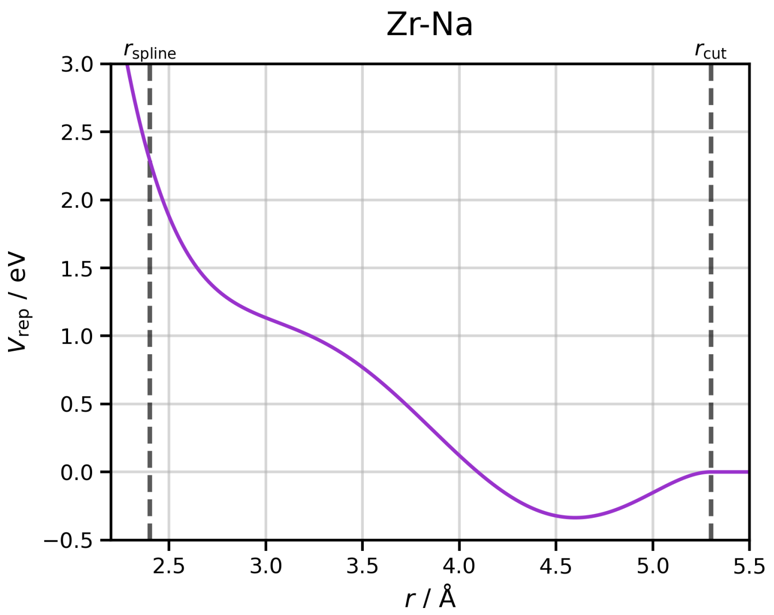

Figure S10: Optimized repulsive potential $V_{\rm rep}$ for the zirconium-sodium interaction. Following the original 3OB parametrization strategy, the potential is described *via* an exponential function for distances below $r_{\rm spline}$. In the range between $r_{\rm spline}$ and $r_{\rm cut}$, the curve is defined by the spline-based protocol. For interatomic distances greater than $r_{\rm cut}$, $V_{\rm rep}$ is set to zero.

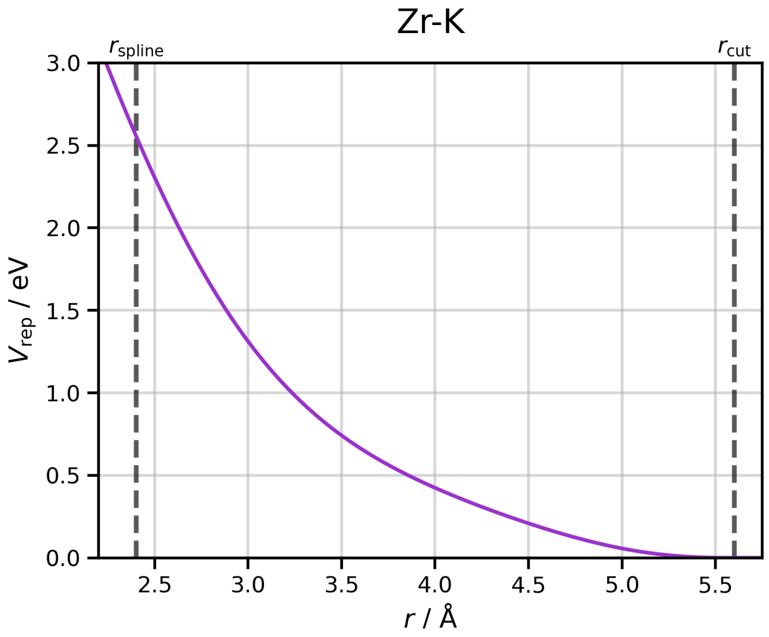

Figure S11: Optimized repulsive potential $V_{\rm rep}$ for the zirconium-potassium interaction. Following the original 3OB parametrization strategy, the potential is described *via* an exponential function for distances below $r_{\rm spline}$. In the range between $r_{\rm spline}$ and $r_{\rm cut}$, the curve is defined by the spline-based protocol. For interatomic distances greater than $r_{\rm cut}$, $V_{\rm rep}$ is set to zero.

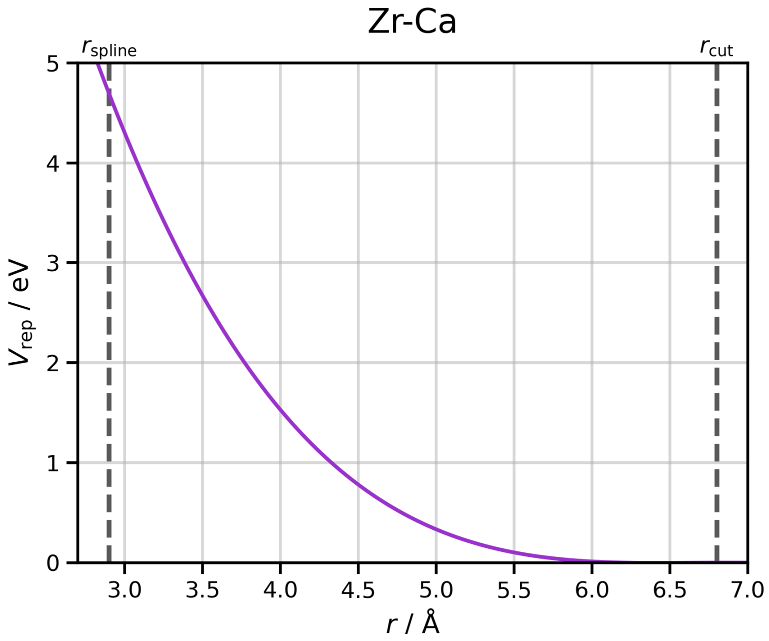

Figure S12: Optimized repulsive potential $V_{\rm rep}$ for the zirconium-calcium interaction. Following the original 3OB parametrization strategy, the potential is described *via* an exponential function for distances below $r_{\rm spline}$. In the range between $r_{\rm spline}$ and $r_{\rm cut}$, the curve is defined by the spline-based protocol. For interatomic distances greater than $r_{\rm cut}$, $V_{\rm rep}$ is set to zero.

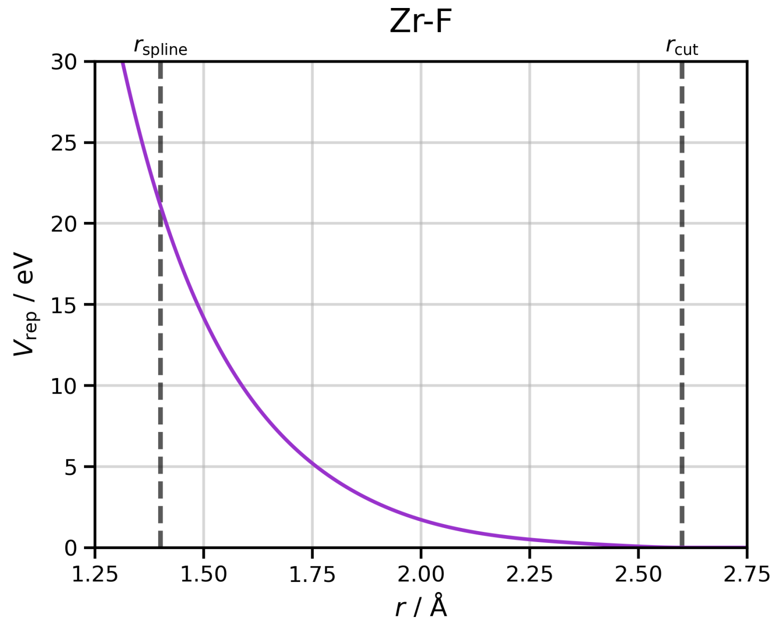

Figure S13: Optimized repulsive potential V_{rep} for the zirconium-fluorine interaction. Following the original 3OB parametrization strategy, the potential is described *via* an exponential function for distances below r_{spline}. In the range between r_{spline} and r_{cut}, the curve is defined by the spline-based protocol. For interatomic distances greater than r_{cut}, V_{rep} is set to zero.

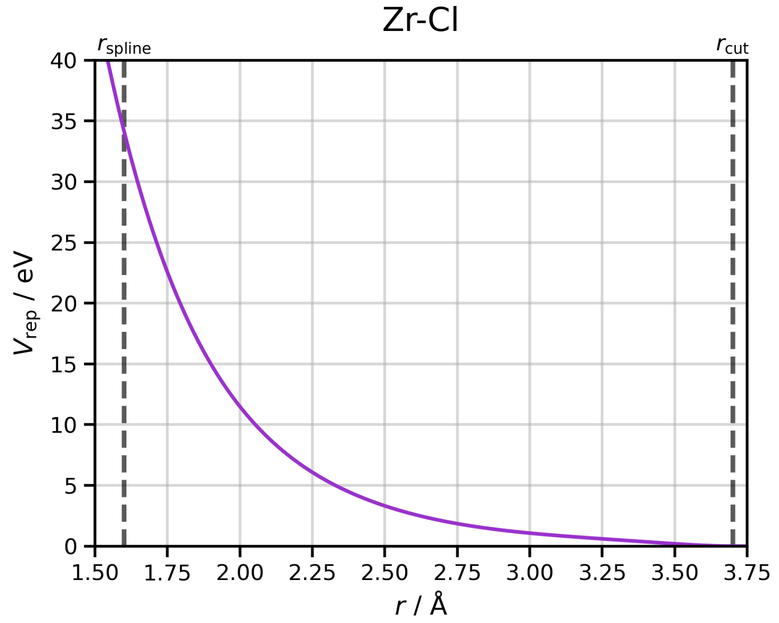

Figure S14: Optimized repulsive potential V_{rep} for the zirconium-chlorine interaction. Following the original 3OB parametrization strategy, the potential is described *via* an exponential function for distances below r_{spline}. In the range between r_{spline} and r_{cut}, the curve is defined by the spline-based protocol. For interatomic distances greater than r_{cut}, V_{rep} is set to zero.

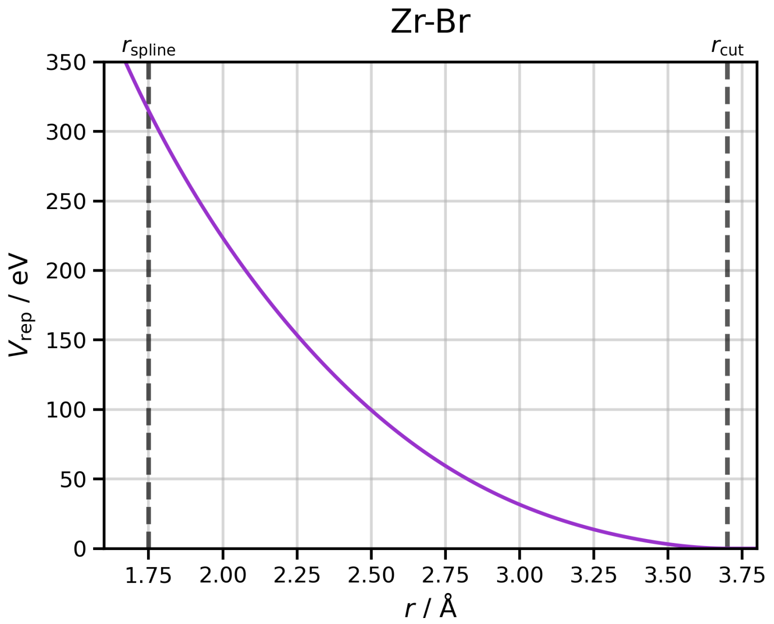

Figure S15: Optimized repulsive potential V_{rep} for the zirconium-bromine interaction. Following the original 3OB parametrization strategy, the potential is described *via* an exponential function for distances below r_{spline}. In the range between r_{spline} and r_{cut}, the curve is defined by the spline-based protocol. For interatomic distances greater than r_{cut}, V_{rep} is set to zero.

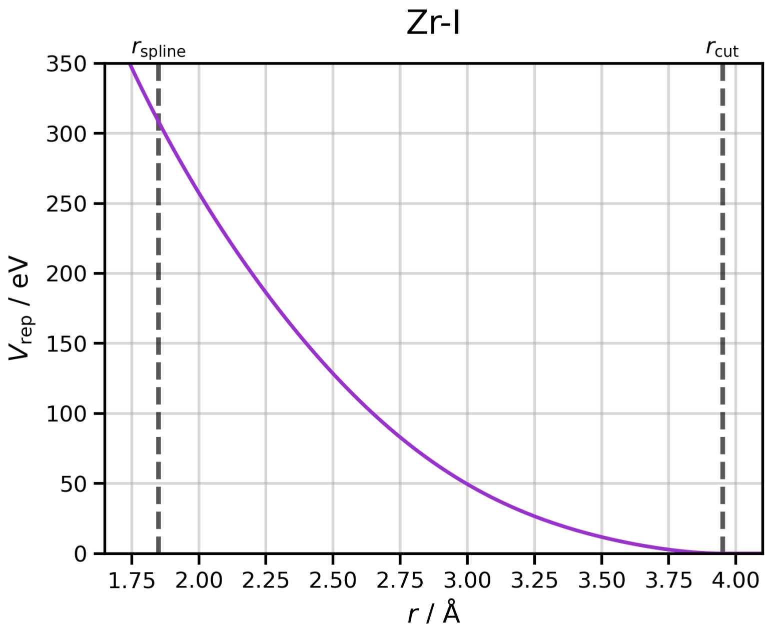

Figure S16: Optimized repulsive potential V_{rep} for the zirconium-iodine interaction. Following the original 3OB parametrization strategy, the potential is described *via* an exponential function for distances below r_{spline}. In the range between r_{spline} and r_{cut}, the curve is defined by the spline-based protocol. For interatomic distances greater than r_{cut}, V_{rep} is set to zero.

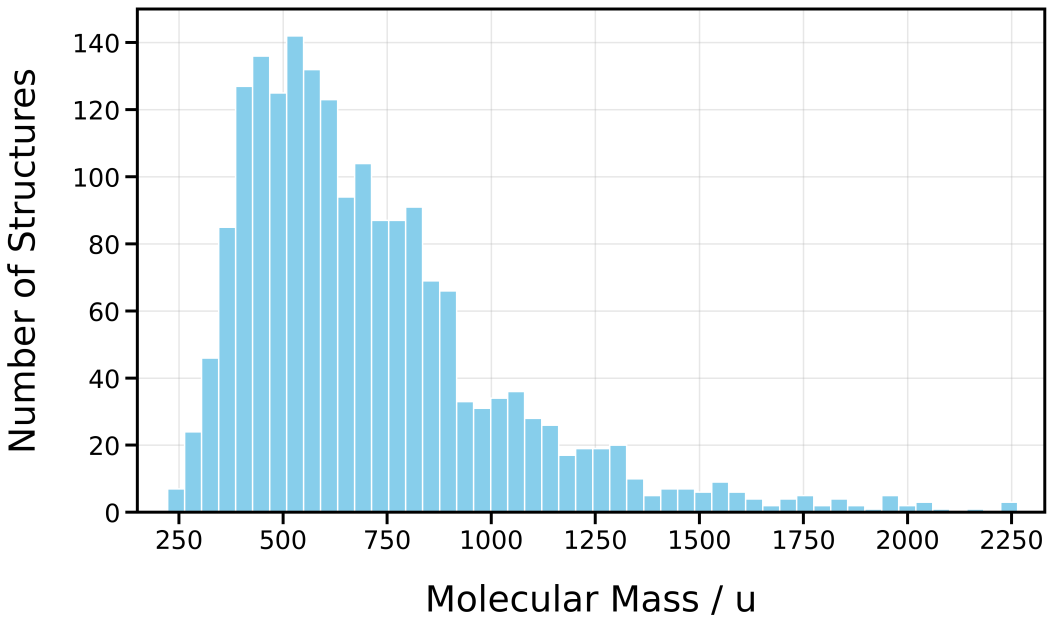

Figure S17: Molecular mass distribution of the 1,897 structures taken from the Cambridge Structural Database for the energy minimizations and subsequent RMSD calculations. The histogram has been generated using a total of 50 bins.

Table S1: List of the 108 Boxplot Outliers for the RMSD Calculation Between the Structures Optimized at DFTB3/3OB Level of Theory and the Structures Deposited in the CSD

CSD ID	RMSD / Å	Molecular Formula	Mass / u
ABUTIB	0.3939	$ZrC_{20}H_{31}OP$	409.665
ABUTOH	0.3908	$ZrC_{17}H_{25}OP$	367.584
ACACZR02	0.4222	$ZrC_{20}H_{28}O_8$	487.660
ACACZR04	0.4102	$ZrC_{20}H_{28}O_8$	487.660
ACOPEP	0.6512	$ZrC_{30}ClH_{29}NP$	561.217
ANOJEV	0.3541	$Zr_4C_{88}Cl_4H_{108}O_4$	1736.524
ANOKEW	0.3771	$ZrBr_2C_{30}H_{38}$	649.666
APEXOK	0.3605	$Zr_4C_{60}H_{76}N_8O_{14}$	1498.206
APOSIJ	0.4150	$ZrC_{30}Cl_2H_{41}O_2P$	626.754
AROPUU	0.3828	$Zr_2C_{56}Cl_8H_{52}N_4O_2$	1279.106
ARUBAS	0.3734	$ZrC_{45}Cl_2H_{62}N_4O_4$	885.139
BAMQEM	0.5178	$ZrC_{21}H_{25}P$	399.629
BEDXAK	0.4379	$ZrC_{22}H_{40}P_2$	457.734
BEDXOA	0.3528	$ZrC_{32}F_{12}H_{40}O_{12}$	935.865
BEPXIE	0.4835	$Zr_2C_{42}Cl_4H_{54}N_2O$	927.155
BIJVEW	0.3696	$ZrC_{13}H_{16}I_2$	517.304
BIKTAR	0.5261	$ZrC_{26}F_{12}H_{28}N_2O_2$	719.727
BIMYEC	0.3815	$Zr_2C_{28}Cl_2H_{38}P_2$	689.908
BINHUC	0.3535	$ZrBr_4C_{20}H_{32}P_4$	807.211
BOJSUQ	0.3713	$ZrC_{46}H_{48}N_2O$	736.127
BZDTZR10	0.4126	$ZrC_{22}H_{24}NS_6$	586.025
CAFNUT	0.4375	$ZrC_{20}Cl_6H_{25}O_8$	697.336
CPDZRB	0.4245	$ZrC_{16}H_{20}$	303.560

Continued on next page

Table S1 continued

CSD ID	RMSD / Å	Molecular Formula	Mass / u
CPDZRB10	0.4235	$ZrC_{16}H_{20}$	303.560
CPDZRB20	0.4240	$ZrC_{16}H_{20}$	303.560
CPZROX04	0.3753	$Zr_2C_{20}Cl_2H_{20}O$	529.727
DAWMIX	0.4568	$ZrC_{34}H_{44}P_2$	605.898
DIJRIZ	0.4451	$Zr_2C_{80}H_{96}O_6S_4$	1464.330
DONBUE	0.3568	$ZrC_{53}H_{64}O_2S$	856.377
ELAPUD01	0.5612	$ZrC_{36}H_{24}N_4O_4$	667.836
EPIJOE	0.5174	$ZrC_{44}H_{73}N_3O_4$	799.309
EPIJUK	0.4048	$ZrC_{45}H_{78}N_2O_5$	818.352
FAGHUQ	0.4360	$Zr_2C_{38}H_{53}P_2$	754.238
FAGKUT	0.4095	$ZrC_{19}H_{23}P$	373.591
FANKEN	0.5328	$ZrC_{29}H_{27}P$	497.733
FEWWOT	0.4549	$Zr_2C_{38}H_{58}P_2$	759.278
FIBZIA	0.3773	$ZrC_{80}H_{78}N_4P_2$	1248.704
HATDEN	0.4044	$ZrC_{30}H_{48}N_4O_2$	587.964
HOPWEQ	0.3884	$ZrC_{34}H_{46}$	545.966
HUCPEB	0.5135	$ZrC_{54}Cl_4H_{46}O_2P_4$	1083.879
IKUKAA	0.3954	$ZrC_{22}H_{42}N_2O_6$	521.810
JAYMAX	0.4767	$ZrC_{23}ClH_{41}P_2$	506.203
JEQBUF	0.3995	$Zr_4C_{74}H_{96}$	1350.478
JEQCAM	0.4182	$Zr_4C_{72}H_{92}$	1322.424
JIMCUD	0.4462	$ZrC_{35}H_{43}N$	568.960
JOFJOF	0.3714	$ZrC_{17}H_{18}N_2O_{11}$	517.558
KAWJIF	0.5428	$Zr_2C_{20}H_{44}N_4O_{10}$	683.038
KOMROV	0.4208	$Zr_2C_{75}H_{98}N_2O_8$	1338.063
KOXHOU	0.3612	$Zr_2C_{32}H_{30}P_2$	658.988
LAHVOG	0.4892	$ZrC_{54}H_{66}O_3S$	886.403
LAHVUM	0.4199	$Zr_2C_{104}H_{126}O_7S_2$	1734.713
LAWRIK	0.4901	$ZrC_{25}H_{29}P$	451.705
MAZKII	0.4520	$ZrC_{35}H_{43}NO_2$	600.958
MIRFAV	0.4097	$ZrC_{32}F_{12}H_{40}O_8$	871.869
MODMIE	0.3971	$ZrC_{44}H_{74}N_2O_4$	786.310
NEGPEV	0.4369	$ZrBr_3C_{14}H_{27}P_2$	588.254
NEJMEU	0.4969	$ZrC_{30}H_{34}N_2O_2$	545.838
OCIYAD	0.5397	$ZrC_{48}H_{70}N_2O_4$	830.322
OGOGOG	0.3526	$Zr_2C_{42}H_{68}OP_4$	895.348
OQIFEA	0.5058	$ZrC_{34}Cl_2H_{34}$	604.770
PAPVUY	0.5866	$ZrC_{24}H_{46}N_2O_6$	549.864
PEDROI	0.3635	$Zr_2C_{34}H_{42}$	633.158
PEYBOK	0.4230	$ZrC_{25}H_{29}P$	451.705
POBSOO	0.3937	$Zr_2C_{40}H_{60}P$	754.342
POJFID	0.4509	$K_2ZrC_{66}H_{96}N_4O_{12}$	1306.931
POVLES	0.4008	$ZrC_{50}H_{41}P_2$	795.050
PUBTEL	0.3732	$Zr_2C_{22}F_6H_{20}O_7S_2$	756.953
RAKRED	0.4586	$ZrC_{57}H_{81}NO_2P_2$	965.452
RAPCOD	0.5174	$ZrC_{58}H_{74}O_4P_2$	988.398
RASLAZ	0.4127	$Zr_2C_{32}H_{44}I_4$	1118.770
RITQAN	0.4737	$ZrC_{65}H_{74}O_2P_2$	1040.477
RURQAW	0.5325	$Zr_2C_{16}Cl_6H_{22}O_2$	641.498
SEFCIP	0.3665	$ZrC_{20}H_{27}P$	389.634
SEJSEG	0.3611	$Zr_2C_{40}H_{80}O_{10}$	903.518

Continued on next page

Table S1 continued

CSD ID	RMSD / Å	Molecular Formula	Mass / u
SEKDUH	0.5005	$ZrC_{19}H_{26}P$	376.615
SICMUM	0.3707	$Zr_2C_{32}H_{28}S_2$	659.144
SIFGUL	0.3613	$ZrC_{58}H_{67}NO_2$	901.403
SIXPAQ	0.3715	$ZrC_{25}H_{42}N_2$	461.849
SULGUB	0.5649	$ZrC_{20}H_{29}P$	391.650
TALSEE	0.4213	$ZrC_{16}H_{16}$	299.528
TALSEE01	0.4127	$ZrC_{16}H_{16}$	299.528
TOBKOL	0.4137	$ZrC_{27}F_{12}H_{24}O_8$	795.686
UDIPAA	0.4081	$ZrC_{24}H_{26}$	405.696
UGIFEV	0.3649	$ZrC_{11}H_{10}I_2$	487.234
UROSUQ	0.3708	$ZrBr_4C_{31}Cl_2H_{28}O_2S_2$	978.423
UWEWUP	0.3748	$Zr_2C_{30}H_{38}P_2$	643.030
VAKYAH01	0.4149	$ZrC_{15}H_{23}P$	325.547
VARJON	0.3991	$ZrC_{21}H_{27}P$	401.645
VAXXOI	0.3668	$ZrC_{24}Cl_2H_{28}N_2O_4$	570.622
VEJVOV	0.4451	$Zr_2C_{34}H_{54}P_2$	707.202
VILNUB	0.3847	$Zr_4C_{91}H_{132}N_8P_4$	1826.904
VIQGIN	0.3745	$ZrC_{36}Cl_2H_{45}O_2P$	702.852
VURKIC	0.4312	$Zr_2C_6Cl_8H_{18}P_2$	618.206
WOFGIH	0.4219	$Zr_6C_{98}H_{106}O_2$	1863.268
WOSFIT	0.3712	$Zr_2C_{68}Cl_4H_{52}$	1193.412
WUGTIC	0.3659	$Zr_2C_{85}H_{118}N_2O_8$	1478.333
WUGTUO	0.4169	$Zr_2C_{92}H_{140}N_2O_8$	1584.586
XILYIA	0.5163	$Zr_2C_{36}H_{40}O_6S_2$	815.278
XOJWOL	0.3724	$ZrC_4Cl_6H_{12}N$	378.071
XOQWUV	0.4549	$ZrC_{51}H_{75}NO_3S$	873.449
XOSNIC	0.4542	$ZrC_{20}H_{36}P_2$	429.680
XOVDER	0.3713	$Zr_2C_{10}Cl_8H_{24}S_4$	738.590
XOVFET	0.5324	$Zr_2C_3Cl_{11}H_8S$	648.555
XOVGUL	0.3663	$ZrC_{15}H_{20}P$	322.523
YUHXOR	0.3826	$ZrC_{57}H_{50}N_4$	882.279
ZAZZOR	0.4001	$ZrC_{15}ClH_{25}O_8$	460.031
ZEXBIN	0.4641	$Zr_2C_{32}H_{38}$	605.104
ZIHZUO	0.3924	$Zr_2C_{77}H_{118}N_2O_8$	1382.245

Table S2: List of the 67 Boxplot Outliers for the RMSD Calculation Between the Structures Optimized at DFTB2/PTBP Level of Theory and the Structures Deposited in the CSD

CSD ID	RMSD / Å	Molecular Formula	Mass / u
ABETUW	0.4292	$Zr_2C_{42}H_{44}$	731.262
APEXIE	0.5047	$Zr_2C_{40}H_{67}N_3O_8$	900.437
BICZUJ	0.6454	$Zr_2C_{28}H_{56}O_{10}$	735.194
BICZUJ01	0.5951	$Zr_2C_{28}H_{56}O_{10}$	735.194
BZDTZR10	0.4242	$ZrC_{22}H_{24}NS_6$	586.025
CALTUE	0.4191	$Zr_2C_{32}H_{76}N_4O_8$	827.428
DAQFOR01	0.4124	$ZrC_{21}H_{21}N$	378.630
EDIKAF	0.4924	$Zr_2C_{89}H_{144}N_2O_{10}$	1584.583
ETAVEA	0.5156	$ZrC_{38}H_{46}N_6O_2$	710.050
FIBZIA	0.5061	$ZrC_{80}H_{78}N_4P_2$	1248.704

Continued on next page

Table S2 continued

CSD ID	RMSD / Å	Molecular Formula	Mass / u
FINNAR	0.4728	$ZrC_{23}H_{28}NP$	440.682
FUBSUR	0.4078	$Zr_4C_{116}H_{176}N_8$	2047.636
GAPKUG	0.4311	$ZrC_{44}Cl_2H_{72}O_2S_2$	859.302
GIXMEF	0.5032	$ZrC_{68}Cl_8H_{84}N_4O_{16}$	1588.256
GIXMIJ	0.4197	$ZrC_{70}ClH_{74}N_4O_8$	1226.056
HAGYOD	0.4283	$NaZrC_{24}F_{36}H_{12}O_6$	1194.510
HILJUH	0.5133	$ZrC_{20}H_{40}N_4O_8$	555.784
HIRBAL	0.4591	$Zr_4C_{76}H_{128}O_{20}$	1726.736
HUCTIK	0.5763	$ZrC_{21}Cl_2H_{34}N_4O_8$	632.647
HURWIB	0.4250	$ZrC_{101}H_{108}N_4O_4$	1533.223
JAQTAW	0.5078	$Zr_2C_{48}H_{48}N_4$	863.388
KAWKUP	0.4446	$Zr_4C_{32}H_{72}O_{16}$	1077.808
KOJLIE	0.4179	$Zr_2C_{32}H_{36}O_{12}$	795.076
LISPOS	0.4743	$ZrC_{22}ClH_{27}O$	434.131
LUZDEP	0.4441	$Zr_2C_{46}H_{88}N_4$	879.686
NOVJIR	0.4176	$ZrC_{48}H_{40}N_{12}$	876.156
OCEWUO	0.4327	$Zr_2C_{75}H_{92}N_4O_2$	1264.035
OQIFEA	0.5110	$ZrC_{34}Cl_2H_{34}$	604.770
POJFID	0.5168	$K_2ZrC_{66}H_{96}N_4O_{12}$	1306.931
PUBTEL	0.4942	$Zr_2C_{22}F_6H_{20}O_7S_2$	756.953
PUQZOQ	0.5403	$ZrC_{30}H_{56}N_4$	564.030
QAJGEO	0.4262	$Zr_2C_{66}H_{72}N_4$	1103.778
QAMPOM	0.4232	$ZrC_{52}H_{60}O_2$	808.274
QAXWOB	0.4547	$Zr_2C_{52}H_{78}O_{11}P_2$	1123.581
RENWIR	0.4283	$Zr_2C_{25}Cl_4H_{52}O_{11}$	852.928
RENWOX	0.4703	$Zr_2C_{25}Cl_6H_{50}O_{11}$	921.812
REXNAM	0.4170	$Zr_2C_{88}H_{152}O_{24}$	1776.608
RIRMOX	0.4262	$Zr_2C_{116}H_{136}N_{16}O_9$	2080.915
RIRPAK	0.4627	$Zr_2C_{124}H_{168}N_4O_6$	1993.178
RIWFUZ	0.4277	$Zr_4C_{84}H_{156}N_8$	1643.124
RIWGAG	0.4141	$Zr_2C_{40}H_{74}N_4$	793.508
SAMSUV	0.4068	$Zr_2C_{72}Cl_2H_{64}P_4$	1306.547
SEJSEG	0.5619	$Zr_2C_{40}H_{80}O_{10}$	903.518
SILBAR	0.4386	$Zr_2C_{36}H_{82}N_2O_8$	853.506
SINGII	0.4126	$ZrC_{28}H_{56}N_4S_8$	796.488
TOFKAC	0.4835	$Zr_2C_{107}Cl_4H_{118}N_2O_6$	1852.377
TUTKUQ	0.4356	$Zr_4C_{74}H_{112}N_4O_{14}$	1646.620
TUXZEU	0.4193	$ZrC_{24}H_{27}N$	420.711
ULUYOP	0.4779	$Zr_2C_{56}H_{90}O_{12}P_2$	1199.720
ULUZEG	0.4957	$Zr_2C_{48}H_{74}O_{14}P_2$	1119.502
UZIREC	0.5195	$Zr_2C_{56}H_{112}N_8O_8$	1208.008
VURKEY	0.5328	$Zr_2C_{54}Cl_8H_{96}O_3P_2$	1321.355
VUYJUW	0.4330	$Zr_2C_{44}ClH_{47}O_3$	841.755
WAGXEI	0.5129	$Zr_4C_{80}Cl_2H_{70}O_{22}$	1819.214
WOSFIT	0.5319	$Zr_2C_{68}Cl_4H_{52}$	1193.412
WUFBIL	0.4976	$ZrC_{16}H_{36}N_8$	431.744
XEVDUZ	0.4372	$ZrC_{37}H_{66}O_7$	714.152
XEVTAX	0.4418	$ZrC_{60}Cl_2H_{82}N_4$	1021.468
XIZCUE	0.5008	$ZrC_{18}H_{40}O_6$	443.736
XOFMEK	0.4068	$Zr_2C_{94}H_{62}N_8O_4$	1550.030
XOGHOQ	0.4588	$ZrC_{130}H_{98}N_8$	1863.494

Continued on next page

Table S2 continued

CSD ID	RMSD / Å	Molecular Formula	Mass / u
XOVDER	0.4251	$Zr_2C_{10}Cl_8H_{24}S_4$	738.590
XUNNEZ	0.4566	$ZrC_{35}Cl_2H_{42}N_4$	680.873
YAMZOE	0.5098	$Zr_2C_{68}H_{104}N_2O_8$	1260.034
YAYDIM	0.4902	$ZrC_{60}H_{44}N_8$	968.292
YIXCUD	0.4323	$Zr_2C_{52}H_{68}$	875.564
ZEKZUN	0.4555	$Zr_4C_{107}H_{120}$	1771.033

Table S3: List of the 125 Boxplot Outliers for the RMSD Calculation Between the Structures Optimized at GFN2-xTB Level of Theory and the Structures Deposited in the CSD

CSD ID	RMSD / Å	Molecular Formula	Mass / u
ACACZR02	0.4795	$ZrC_{20}H_{28}O_8$	487.660
ACACZR04	0.4764	$ZrC_{20}H_{28}O_8$	487.660
ADOCUT	0.3488	$ZrC_{19}H_{26}$	345.641
ANOJEV	0.4068	$Zr_4C_{88}Cl_4H_{108}O_4$	1736.524
BEDMIH	0.3474	$Zr_2C_{40}H_{72}N_{16}$	959.576
BEXDUG	0.5626	$ZnZrC_{46}H_{64}N_2$	801.636
BIJTUK	0.3735	$Zr_2C_{26}Cl_4H_{32}$	668.790
BIJVAS	0.3826	$ZrBr_2C_{13}H_{16}$	423.303
BIJVEW	0.4123	$ZrC_{13}H_{16}I_2$	517.304
BIMYEC	0.4025	$Zr_2C_{28}Cl_2H_{38}P_2$	689.908
BINHUC	0.3581	$ZrBr_4C_{20}H_{32}P_4$	807.211
BOJSUQ	0.3489	$ZrC_{46}H_{48}N_2O$	736.127
CAJTUC	0.3427	$ZrC_{31}H_{34}N_2O_4$	589.847
CALVET	0.3835	$Zr_2C_{52}H_{84}P_2S_4$	1081.880
CAMZAU	0.3421	$Zr_4C_{46}Cl_2H_{82}$	1070.958
CEPTIE	0.3995	$Zn_2Zr_2C_{30}H_{64}O_{14}$	962.036
CIPGAK	0.3724	$ZrBr_2C_{43}H_{56}N_2O_4$	915.963
CPDCZR03	0.3973	$Zr_2C_{20}Cl_4H_{20}$	584.628
CPDCZR04	0.4061	$Zr_2C_{20}Cl_4H_{20}$	584.628
CPDZRB	0.4910	$ZrC_{16}H_{20}$	303.560
CPDZRB10	0.4918	$ZrC_{16}H_{20}$	303.560
CPDZRB20	0.4904	$ZrC_{16}H_{20}$	303.560
CPZRBZ	0.5108	$ZrC_{18}H_{18}$	325.566
CPZRBZ10	0.5096	$ZrC_{18}H_{18}$	325.566
CPZROX04	0.5745	$Zr_2C_{20}Cl_2H_{20}O$	529.727
DAKMOR	0.4176	$ZrC_{20}H_{24}$	355.636
DIJRIZ	0.4453	$Zr_2C_{80}H_{96}O_6S_4$	1464.330
DUBDAF	0.5609	$Zr_2C_{46}H_{40}O_2$	807.272
EJINUJ	0.4724	$ZrC_{17}H_{25}P_3$	413.532
EPUVIX	0.3900	$ZrC_{38}H_{38}N_2$	613.960
ERIJAQ	0.4071	$Zr_2C_{36}H_{52}N_2$	695.274
ERIJEU	0.5821	$Zr_2C_{36}H_{56}N_2$	699.306
FANKEN	0.5091	$ZrC_{29}H_{27}P$	497.733
FIBZIA	0.4211	$ZrC_{80}H_{78}N_4P_2$	1248.704
FIFBOL	0.3532	$ZrC_{10}H_{11}$	222.422
FOSLOO	0.3428	$ZrBr_2C_{16}H_{26}$	469.416
FUNSUD	0.3688	$ZrC_{18}Cl_2H_{24}N_2$	430.528
GANCAC	0.0373	$ZrC_{24}H_{48}N_4O_4$	547.896

Continued on next page

Table S3 continued

CSD ID	RMSD / Å	Molecular Formula	Mass / u
GAPWIE	0.3735	$Zr_2C_{19}Cl_{10}H_{18}O_2$	815.299
GIKFEL	0.4279	$Zr_2C_{22}ClH_{26}P$	539.322
HABLAX03	0.3580	$Zr_2C_8F_8H_{24}O_4S_4$	646.951
HEFRER	0.3508	$Zr_4C_{36}Cl_{12}H_{52}$	1275.108
HEYXUE	0.4368	$Zr_2C_{34}Cl_2H_{54}$	716.154
HIGPIW	0.5255	$Zr_2C_{40}H_{44}O_2$	739.238
HOBYAY	0.3778	$ZrC_{16}Cl_2H_{22}$	376.476
HUCTIK	0.4906	$ZrC_{21}Cl_2H_{34}N_4O_8$	632.647
IKUKAA	0.4188	$ZrC_{22}H_{42}N_2O_6$	521.810
IRIVAH	0.3931	$Zr_2C_{40}H_{56}$	719.336
IVAVIK	0.3500	$Zr_2C_{62}H_{54}P_2S_2$	1107.630
JAYCUI	0.3490	$ZrBr_2C_{13}H_{14}$	421.287
JEQBUF	0.3601	$Zr_4C_{74}H_{96}$	1350.478
JEQCAM	0.3734	$Zr_4C_{72}H_{92}$	1322.424
JIMCUD	0.4796	$ZrC_{35}H_{43}N$	568.960
JIVKOO	0.3506	$Zr_2C_{76}H_{68}N_4$	1219.856
JOFJIZ	0.4752	$ZrC_{23}H_{27}N_5O_{11}$	640.717
KESXAH	0.4982	$Zr_2C_{16}Cl_8H_{32}O_4$	754.476
KIFTOM	0.4543	$ZrC_{24}H_{36}N_4O_4$	535.800
LAHVOG	0.3731	$ZrC_{54}H_{66}O_3S$	886.403
LAHVUM	0.4298	$Zr_2C_{104}H_{126}O_7S_2$	1734.713
LAWRIK	0.3971	$ZrC_{25}H_{29}P$	451.705
LEMLIY	0.3772	$ZrC_{22}ClH_{21}$	412.084
LULMAI	0.3619	$Zr_2C_{48}Cl_2H_{50}O_8$	1008.268
MAPJIW	0.3597	$ZrC_{40}H_{42}N_2$	642.014
MAVWAJ	0.6062	$ZrC_{33}Cl_2H_{32}O_2P_2S_2$	748.809
MIRFEZ	0.5262	$ZrC_{20}F_{12}H_{16}O_8$	703.545
NEDKOW	0.4138	$ZrC_{45}ClH_{33}O_6$	796.427
NEGPEV	0.3924	$ZrBr_3C_{14}H_{27}P_2$	588.254
NUNQOC	0.3654	$ZrC_{44}H_{60}$	680.188
OGOGOG	0.3861	$Zr_2C_{42}H_{68}OP_4$	895.348
OQIFEA	0.5281	$ZrC_{34}Cl_2H_{34}$	604.770
OWEWIY	0.3468	$ZrC_{20}ClH_{25}$	392.094
OWEWOE	0.3525	$ZrC_{23}ClH_{31}$	434.175
OWEXEV	0.3430	$ZrC_{23}ClH_{25}$	428.127
PAJJOB	0.4242	$Zr_2C_{48}Cl_6H_{72}N_2P_4$	1196.161
PAYLAD	0.4151	$Zr_2C_{26}Cl_6H_{44}P_2$	813.734
PAYLOR	0.3751	$Zr_2C_{18}Cl_6H_{28}P_2$	701.518
PEDROI	0.3489	$Zr_2C_{34}H_{42}$	633.158
PIWZED	0.3717	$ZrC_{54}H_{36}N_{10}O_3$	964.173
POBSOO	0.3405	$Zr_2C_{40}H_{60}P$	754.342
POVLES	0.3831	$ZrC_{50}H_{41}P_2$	795.050
QAGCUY	0.3715	$ZrC_{40}H_{54}O_2$	658.094
QAMPOM	0.3720	$ZrC_{52}H_{60}O_2$	808.274
QAWBIA	0.3509	$Zr_2C_{36}H_{52}N_2$	695.274
RASLAZ	0.4542	$Zr_2C_{32}H_{44}I_4$	1118.770
RIZZIK	0.4749	$Zr_2C_{46}Cl_6H_{46}N_4$	1050.050
ROCLIG	0.3808	$ZrC_{44}H_{44}O$	680.059
RURQAW	0.5851	$Zr_2C_{16}Cl_6H_{22}O_2$	641.498
SEKDUH	0.3567	$ZrC_{19}H_{26}P$	376.615
SICMUM	0.3573	$Zr_2C_{32}H_{28}S_2$	659.144

Continued on next page

Table S3 continued

CSD ID	RMSD / Å	Molecular Formula	Mass / u
SIPKAE	0.3409	$ZrC_{24}H_{28}N_4$	463.740
SIXPAQ	0.3721	$ZrC_{25}H_{42}N_2$	461.849
SUNSEB	0.3891	$ZrC_{23}H_{31}N$	412.732
TALSEE	0.4249	$ZrC_{16}H_{16}$	299.528
TALSEE01	0.4296	$ZrC_{16}H_{16}$	299.528
TOBKOL	0.4215	$ZrC_{27}F_{12}H_{24}O_8$	795.686
UDIPAA	0.3807	$ZrC_{24}H_{26}$	405.696
UFIDOF	0.3546	$Zr_2C_{44}Cl_6H_{84}N_2P_4$	1160.213
UGIFEV	0.4026	$ZrC_{11}H_{10}I_2$	487.234
UHEJAS	0.4030	$Zr_2C_{52}Cl_4H_{52}$	1001.236
UPOJEP	0.4071	$ZrC_{58}H_{62}O_4$	914.354
UTABOI	0.3954	$ZrC_{24}H_{54}N_4O_2P_2S_2$	648.014
UWEWUP	0.3544	$Zr_2C_{30}H_{38}P_2$	643.030
VEBLOD	0.5963	$Zr_2C_8Cl_8H_{16}O_4$	642.260
VEWPAO	0.4698	$Zr_3C_{45}H_{57}P_3$	964.544
VILNUB	0.3396	$Zr_4C_{91}H_{132}N_8P_4$	1826.904
VOSVAA	0.3665	$Zr_2C_{26}Cl_4H_{52}P_4$	812.845
VOSVAA10	0.3678	$Zr_2C_{26}Cl_4H_{52}P_4$	812.845
WOFGIH	0.3993	$Zr_6C_{98}H_{106}O_2$	1863.268
WUPVUA	0.3809	$Zn_2Zr_2C_{44}H_{72}O_2$	946.266
XELQAK	0.3715	$Zr_2C_{58}Cl_2H_{66}N_4$	1072.542
XELQEO	0.3847	$Zr_2C_{44}Cl_2H_{70}N_4$	908.420
XILYIA	0.5061	$Zr_2C_{36}H_{40}O_6S_2$	815.278
XIWHAN	0.4316	$ZrC_{51}H_{48}NP$	797.150
XIZCUE	0.3757	$ZrC_{18}H_{40}O_6$	443.736
XOJWOL	0.3449	$ZrC_4Cl_6H_{12}N$	378.071
XOVFET	0.5168	$Zr_2C_3Cl_{11}H_8S$	648.555
XOVGUL	0.3586	$ZrC_{15}H_{20}P$	322.523
YOGKAH	0.3441	$Zr_2C_{56}H_{66}N_8O$	1049.647
YUHXOR	0.3588	$ZrC_{57}H_{50}N_4$	882.279
ZASDII	0.3600	$Zr_3C_{16}H_{24}S_{18}$	1067.120
ZEFZOZ	0.6158	$ZrC_{26}H_{24}$	427.702
ZEGJUS	0.3876	$Zr_2C_{68}Cl_4H_{94}N_4O_2$	1323.774
ZEXBIN	0.3929	$Zr_2C_{32}H_{38}$	605.104
ZIQCUX	0.4732	$Zr_2C_{72}H_{76}N_4O_8$	1307.868
ZUJLOF	0.3943	$Zr_2Br_4C_{20}H_{20}$	762.444

Table S4: List of the 37 Boxplot Outliers for the RMSD Calculation Between the Structures Optimized at MACE-MP-0 Level of Theory and the Structures Deposited in the CSD

CSD ID	RMSD / Å	Molecular Formula	Mass / u
ADALUM	0.4772	$Zr_2C_{48}Cl_4H_{48}P_4$	1073.055
AROHIA	0.4351	$ZrC_{23}Cl_3H_{19}N_4O$	565.006
BIJTUK	0.5084	$Zr_2C_{26}Cl_4H_{32}$	668.790
CAJTUC	0.4375	$ZrC_{31}H_{34}N_2O_4$	589.847
CIPDOV	0.4917	$ZrC_{22}H_{22}$	377.642
CPACZR02	0.4806	$ZrC_{15}ClH_{19}O_4$	389.987
DIHTEU	0.4378	$ZrC_{24}H_{32}O$	427.743
DUHWAG	0.4652	$ZrC_{24}Cl_2H_{16}$	466.516

Continued on next page

Table S4 continued

CSD ID	RMSD / Å	Molecular Formula	Mass / u
FOSLOO	0.4367	$ZrBr_2C_{16}H_{26}$	469.416
HAMSIX	0.4460	$ZrC_{19}H_{17}NO_4$	414.572
JUSPIZ	0.4432	$ZrC_{18}F_3H_{16}N$	394.552
KEMRIE	0.4386	$ZrC_{33}H_{37}$	524.883
KICRUN	0.4445	$Zr_2C_{32}F_{12}H_{36}$	831.069
LANJAL	0.4408	$Zr_2C_{32}H_{44}O_4$	675.148
LAWRIK	0.4379	$ZrC_{25}H_{29}P$	451.705
NEGPEV	0.4393	$ZrBr_3C_{14}H_{27}P_2$	588.254
NEVDEX	0.4480	$Zr_2C_{44}Cl_4H_{36}$	889.020
OCUKUS	0.4517	$ZrC_{53}Cl_2H_{48}$	847.091
OQIFEA	0.5038	$ZrC_{34}Cl_2H_{34}$	604.770
QAJGOY	0.5228	$ZrC_{20}Cl_2H_{16}$	418.472
QAPHAQ	0.6031	$ZrC_{19}Cl_2H_{14}$	404.445
QAPHAQ01	0.5834	$ZrC_{19}Cl_2H_{14}$	404.445
QAPHEU	0.5045	$ZrC_{23}Cl_2H_{22}$	460.553
QEWPUD	0.4402	$ZrC_{27}Cl_2H_{30}$	516.661
QOJKOR	0.4412	$ZrC_{23}ClH_{45}N_6$	532.329
RIFPAX	0.4533	$ZrC_{18}Cl_2H_{14}$	392.434
SADWOK	0.5383	$ZrC_{18}F_2H_{22}$	367.595
SEHDIS	0.4954	$ZrC_{32}ClH_{32}N_2P$	602.270
SULGUB	0.4300	$ZrC_{20}H_{29}P$	391.650
TALSEE	0.5661	$ZrC_{16}H_{16}$	299.528
TALSEE01	0.5494	$ZrC_{16}H_{16}$	299.528
TIZYIK	0.4936	$ZrC_{24}H_{28}N_2$	435.726
UTUHOI	0.4436	$Zr_4C_{62}H_{88}$	1198.282
VEHLEZ	0.4323	$ZrC_{13}Cl_3H_{21}O_2$	406.883
WOFGIH	0.4473	$Zr_6C_{98}H_{106}O_2$	1863.268
YEFHAS	0.4390	$Zr_2C_{16}Cl_5H_{41}P_6$	779.045
YOTFES01	0.4863	$ZrC_{20}Cl_2H_{16}$	418.472

Table S5: List of the 27 Boxplot Outliers for the RMSD Calculation Between the Structures Optimized at DFTB2/PTBP and DFTB3/3OB Level of Theory

CSD ID	RMSD / Å	Molecular Formula	Mass / u
APEXIE	0.5419	$Zr_2C_{40}H_{67}N_3O_8$	900.437
BAMQEM	0.5016	$ZrC_{21}H_{25}P$	399.629
BEPXIE	0.5024	$Zr_2C_{42}Cl_4H_{54}N_2O$	927.155
BICZUJ	0.5129	$Zr_2C_{28}H_{56}O_{10}$	735.194
BIKTAR	0.6957	$ZrC_{26}F_{12}H_{28}N_2O_2$	719.727
CEPTIE	0.5156	$Zn_2Zr_2C_{30}H_{64}O_{14}$	962.036
DAWMIX	0.5204	$ZrC_{34}H_{44}P_2$	605.898
DIJRIZ	0.5352	$Zr_2C_{80}H_{96}O_6S_4$	1464.330
ELAPUD01	0.5787	$ZrC_{36}H_{24}N_4O_4$	667.836
ETAVEA	0.5046	$ZrC_{38}H_{46}N_6O_2$	710.050
FANKEN	0.5515	$ZrC_{29}H_{27}P$	497.733
HUCTIK	0.5826	$ZrC_{21}Cl_2H_{34}N_4O_8$	632.647
IKUKAA	0.5402	$ZrC_{22}H_{42}N_2O_6$	521.810
KAWJIF	0.5788	$Zr_2C_{20}H_{44}N_4O_{10}$	683.038
LAHVOG	0.5244	$ZrC_{54}H_{66}O_3S$	886.403

Continued on next page

Table S5 continued

CSD ID	RMSD / Å	Molecular Formula	Mass / u
NEJMEU	0.6311	$ZrC_{30}H_{34}N_2O_2$	545.838
OCIYAD	0.5954	$ZrC_{48}H_{70}N_2O_4$	830.322
PAPVUY	0.7476	$ZrC_{24}H_{46}N_2O_6$	549.864
QAXWOB	0.5025	$Zr_2C_{52}H_{78}O_{11}P_2$	1123.581
RAPCOD	0.6758	$ZrC_{58}H_{74}O_4P_2$	988.398
RURQAW	0.5342	$Zr_2C_{16}Cl_6H_{22}O_2$	641.498
SULGUB	0.5371	$ZrC_{20}H_{29}P$	391.650
ULUZEG	0.5686	$Zr_2C_{48}H_{74}O_{14}P_2$	1119.502
WAGXEI	0.5598	$Zr_4C_{80}Cl_2H_{70}O_{22}$	1819.214
WUFBIL	0.4997	$ZrC_{16}H_{36}N_8$	431.744
WUGTUO	0.5298	$Zr_2C_{92}H_{140}N_2O_8$	1584.586
XIZCUE	0.5338	$ZrC_{18}H_{40}O_6$	443.736

Table S6: List of the 96 Boxplot Outliers for the RMSD Calculation Between the Structures Optimized at GFN2-xTB and DFTB3/3OB Level of Theory

CSD ID	RMSD / Å	Molecular Formula	Mass / u
ACACZR02	0.3389	$ZrC_{20}H_{28}O_8$	487.660
ACACZR04	0.3462	$ZrC_{20}H_{28}O_8$	487.660
ACOPEP	0.6558	$ZrC_{30}ClH_{29}NP$	561.217
APEXOK	0.3407	$Zr_4C_{60}H_{76}N_8O_{14}$	1498.206
APOSIJ	0.4213	$ZrC_{30}Cl_2H_{41}O_2P$	626.754
ARALIP	0.3273	$ZrC_{39}Cl_4H_{60}O_2S_2$	858.051
AROPUU	0.4179	$Zr_2C_{56}Cl_8H_{52}N_4O_2$	1279.106
ARUBAS	0.3524	$ZrC_{45}Cl_2H_{62}N_4O_4$	885.139
AWAMAP	0.3310	$ZrC_{32}H_{46}O_2$	553.942
BAMQEM	0.3736	$ZrC_{21}H_{25}P$	399.629
BEDXAK	0.3347	$ZrC_{22}H_{40}P_2$	457.734
BEPXIE	0.4485	$Zr_2C_{42}Cl_4H_{54}N_2O$	927.155
BEXDUG	0.4472	$ZnZrC_{46}H_{64}N_2$	801.636
BIKTAR	0.5803	$ZrC_{26}F_{12}H_{28}N_2O_2$	719.727
BOJSUQ	0.3352	$ZrC_{46}H_{48}N_2O$	736.127
BZDTZR10	0.3979	$ZrC_{22}H_{24}NS_6$	586.025
CAFNUT	0.4151	$ZrC_{20}Cl_6H_{25}O_8$	697.336
CPDCZR03	0.3536	$Zr_2C_{20}Cl_4H_{20}$	584.628
CPDCZR04	0.3612	$Zr_2C_{20}Cl_4H_{20}$	584.628
CPZRBZ	0.4139	$ZrC_{18}H_{18}$	325.566
CPZRBZ10	0.4120	$ZrC_{18}H_{18}$	325.566
DAKMOR	0.3488	$ZrC_{20}H_{24}$	355.636
DAWMIX	0.3884	$ZrC_{34}H_{44}P_2$	605.898
DUBDAF	0.4702	$Zr_2C_{46}H_{40}O_2$	807.272
ELAPUD01	0.5349	$ZrC_{36}H_{24}N_4O_4$	667.836
EPIJOE	0.4957	$ZrC_{44}H_{73}N_3O_4$	799.309
EPIJUK	0.3954	$ZrC_{45}H_{78}N_2O_5$	818.352
ERIJEU	0.5660	$Zr_2C_{36}H_{56}N_2$	699.306
FAGHUQ	0.3425	$Zr_2C_{38}H_{53}P_2$	754.238
FEWWOT	0.3443	$Zr_2C_{38}H_{58}P_2$	759.278
FIBZIA	0.3309	$ZrC_{80}H_{78}N_4P_2$	1248.704
GAPWIE	0.3518	$Zr_2C_{19}Cl_{10}H_{18}O_2$	815.299

Continued on next page

Table S6 continued

CSD ID	RMSD / Å	Molecular Formula	Mass / u
GIQBOA	0.3146	$ZrC_{63}ClH_{72}N_3O_6$	1093.958
GOLSIM01	0.4569	$ZrC_4Cl_6H_{12}N$	378.071
HABLAX03	0.4430	$Zr_2C_8F_8H_{24}O_4S_4$	646.951
HEYXUE	0.3350	$Zr_2C_{34}Cl_2H_{54}$	716.154
HIGPIW	0.5653	$Zr_2C_{40}H_{44}O_2$	739.238
HUCPEB	0.4871	$ZrC_{54}Cl_4H_{46}O_2P_4$	1083.879
HUCTIK	0.4938	$ZrC_{21}Cl_2H_{34}N_4O_8$	632.647
IGOGAO	0.3170	$ZrC_{21}Cl_4H_{34}N_2O_2$	579.539
IRIVAH	0.4114	$Zr_2C_{40}H_{56}$	719.336
JAYMAX	0.5505	$ZrC_{23}ClH_{41}P_2$	506.203
JOFJIZ	0.5854	$ZrC_{23}H_{27}N_5O_{11}$	640.717
JOTGIL	0.3201	$ZrC_{45}Cl_4H_{58}O_3S$	912.040
KAWJIF	0.4354	$Zr_2C_{20}H_{44}N_4O_{10}$	683.038
KESXAH	0.5073	$Zr_2C_{16}Cl_8H_{32}O_4$	754.476
KIFTOM	0.4274	$ZrC_{24}H_{36}N_4O_4$	535.800
KOMROV	0.3524	$Zr_2C_{75}H_{98}N_2O_8$	1338.063
LULMAI	0.3248	$Zr_2C_{48}Cl_2H_{50}O_8$	1008.268
MAPJIW	0.3272	$ZrC_{40}H_{42}N_2$	642.014
MAVWAJ	0.4379	$ZrC_{33}Cl_2H_{32}O_2P_2S_2$	748.809
MAZKII	0.4328	$ZrC_{35}H_{43}NO_2$	600.958
MIRFAV	0.3443	$ZrC_{32}F_{12}H_{40}O_8$	871.869
MIRFEZ	0.4171	$ZrC_{20}F_{12}H_{16}O_8$	703.545
MODMIE	0.3702	$ZrC_{44}H_{74}N_2O_4$	786.310
NANHAN	0.3310	$ZrC_{46}H_{66}O_4S_2$	838.374
NEGPEV	0.3746	$ZrBr_3C_{14}H_{27}P_2$	588.254
NEJMEU	0.5505	$ZrC_{30}H_{34}N_2O_2$	545.838
OCIYAD	0.3810	$ZrC_{48}H_{70}N_2O_4$	830.322
PAPVUY	0.5591	$ZrC_{24}H_{46}N_2O_6$	549.864
POJFID	0.4390	$K_2ZrC_{66}H_{96}N_4O_{12}$	1306.931
QAMPOM	0.4605	$ZrC_{52}H_{60}O_2$	808.274
QEQHUP	0.3723	$ZrC_{36}H_{43}NO_3$	628.968
RAKRED	0.4521	$ZrC_{57}H_{81}NO_2P_2$	965.452
RAPCOD	0.5243	$ZrC_{58}H_{74}O_4P_2$	988.398
RITQAN	0.4944	$ZrC_{65}H_{74}O_2P_2$	1040.477
SEKDUH	0.3580	$ZrC_{19}H_{26}P$	376.615
SIFGUL	0.3375	$ZrC_{58}H_{67}NO_2$	901.403
SULGUB	0.4789	$ZrC_{20}H_{29}P$	391.650
SUNSEB	0.3755	$ZrC_{23}H_{31}N$	412.732
TEPPIQ	0.3152	$ZrC_{63}H_{86}N_2O_2$	994.617
UHEJAS	0.3209	$Zr_2C_{52}Cl_4H_{52}$	1001.236
UROSUQ	0.3923	$ZrBr_4C_{31}Cl_2H_{28}O_2S_2$	978.423
VAKYAH01	0.3457	$ZrC_{15}H_{23}P$	325.547
VAXXOI	0.3272	$ZrC_{24}Cl_2H_{28}N_2O_4$	570.622
VEBLOD	0.5840	$Zr_2C_8Cl_8H_{16}O_4$	642.260
VEJVOV	0.3419	$Zr_2C_{34}H_{54}P_2$	707.202
VEWPAO	0.3351	$Zr_3C_{45}H_{57}P_3$	964.544
VIQGIN	0.3601	$ZrC_{36}Cl_2H_{45}O_2P$	702.852
VOSVAA	0.3180	$Zr_2C_{26}Cl_4H_{52}P_4$	812.845
VOSVAA10	0.3189	$Zr_2C_{26}Cl_4H_{52}P_4$	812.845
VURKIC	0.3321	$Zr_2C_6Cl_8H_{18}P_2$	618.206
WUGTUO	0.3519	$Zr_2C_{92}H_{140}N_2O_8$	1584.586

Continued on next page

Table S6 continued

CSD ID	RMSD / Å	Molecular Formula	Mass / u
XELQAK	0.3685	$Zr_2C_{58}Cl_2H_{66}N_4$	1072.542
XELQEO	0.3263	$Zr_2C_{44}Cl_2H_{70}N_4$	908.420
XILYIA	0.3821	$Zr_2C_{36}H_{40}O_6S_2$	815.278
XIZCUE	0.4045	$ZrC_{18}H_{40}O_6$	443.736
XOJWOL	0.6160	$ZrC_4Cl_6H_{12}N$	378.071
XOQWUV	0.3923	$ZrC_{51}H_{75}NO_3S$	873.449
XOSNIC	0.3272	$ZrC_{20}H_{36}P_2$	429.680
XOVDER	0.3444	$Zr_2C_{10}Cl_8H_{24}S_4$	738.590
XOVFET	0.6894	$Zr_2C_3Cl_{11}H_8S$	648.555
ZAZZOR	0.3853	$ZrC_{15}ClH_{25}O_8$	460.031
ZEFZOZ	0.5013	$ZrC_{26}H_{24}$	427.702
ZIQCUX	0.4343	$Zr_2C_{72}H_{76}N_4O_8$	1307.868
ZOJVAV	0.3526	$Zr_2C_{29}Cl_2F_7H_{44}N$	793.015

Table S7: List of the 34 Boxplot Outliers for the RMSD Calculation Between the Structures Optimized at MACE-MP-0 and DFTB3/3OB Level of Theory

CSD ID	RMSD / Å	Molecular Formula	Mass / u
ACOPEP	0.6524	$ZrC_{30}ClH_{29}NP$	561.217
BAMQEM	0.5661	$ZrC_{21}H_{25}P$	399.629
BAMVIU	0.5684	$Zr_2C_{24}H_{32}$	502.968
BIJTUK	0.6200	$Zr_2C_{26}Cl_4H_{32}$	668.790
EJINUJ	0.5725	$ZrC_{17}H_{25}P_3$	413.532
ELAPUD01	0.6110	$ZrC_{36}H_{24}N_4O_4$	667.836
FANKEN	0.5699	$ZrC_{29}H_{27}P$	497.733
FOSLOO	0.6397	$ZrBr_2C_{16}H_{26}$	469.416
JEQBUF	0.6353	$Zr_4C_{74}H_{96}$	1350.478
JEQCAM	0.6508	$Zr_4C_{72}H_{92}$	1322.424
JEQCEQ	0.5811	$Zr_2C_{36}H_{46}$	661.212
MIRFAV	0.5999	$ZrC_{32}F_{12}H_{40}O_8$	871.869
NEGPEV	0.6412	$ZrBr_3C_{14}H_{27}P_2$	588.254
NEVDEX	0.5886	$Zr_2C_{44}Cl_4H_{36}$	889.020
OCIYAD	0.5679	$ZrC_{48}H_{70}N_2O_4$	830.322
PAPVUY	0.5882	$ZrC_{24}H_{46}N_2O_6$	549.864
PEDROI	0.6228	$Zr_2C_{34}H_{42}$	633.158
QAJGOY	0.5753	$ZrC_{20}Cl_2H_{16}$	418.472
QAPHAQ	0.6407	$ZrC_{19}Cl_2H_{14}$	404.445
QAPHAQ01	0.6174	$ZrC_{19}Cl_2H_{14}$	404.445
QAPHEU	0.5828	$ZrC_{23}Cl_2H_{22}$	460.553
RASLAZ	0.6957	$Zr_2C_{32}H_{44}I_4$	1118.770
SADWOK	0.6169	$ZrC_{18}F_2H_{22}$	367.595
SULGUB	0.7579	$ZrC_{20}H_{29}P$	391.650
TALSEE	0.6680	$ZrC_{16}H_{16}$	299.528
TALSEE01	0.7031	$ZrC_{16}H_{16}$	299.528
TOBKOL	0.6164	$ZrC_{27}F_{12}H_{24}O_8$	795.686
UTUHOI	0.7132	$Zr_4C_{62}H_{88}$	1198.282
WOFGIH	0.5916	$Zr_6C_{98}H_{106}O_2$	1863.268
XILYIA	0.6706	$Zr_2C_{36}H_{40}O_6S_2$	815.278
XOSNIC	0.5723	$ZrC_{20}H_{36}P_2$	429.680

Continued on next page

Table S7 continued

CSD ID	RMSD / Å	Molecular Formula	Mass / u
YEFHAS	0.6208	$Zr_2C_{16}Cl_5H_{41}P_6$	779.045
YOTFES01	0.5811	$ZrC_{20}Cl_2H_{16}$	418.472
ZEXBIN	0.5698	$Zr_2C_{32}H_{38}$	605.104

S1 Benchmark of Ionic Solids

As stated in the article, the newly derived parameters are inadequate for the description of solid state systems with ionic character. This has been demonstrated by carrying out energy minimizations on tetragonal zirconia ZrO_2 (space group no. 137), as well as cubic zirconium carbide ZrC (space group no. 225). The resulting lattice parameters and interatomic distances are summarized and compared to values reported in the literature in Table S8 and Table S9. For ZrO_2, a significant distortion of the c lattice parameter is evident, accompanied by a substantial deviation in zirconium-oxygen distances. In the case of ZrC, the lattice parameter is notably overestimated, while the carbon-carbon distance is drastically underestimated. Furthermore, the rock salt crystal structure type is lost, resulting in different values for the Zr-Zr and C-C distances.

Table S8: Comparison of Structural Parameters of Tetragonal Zirconia ZrO_2

method	$a = b$ / Å	c / Å	Zr-O_{short} / Å	Zr-O_{long} / Å	source
DFTB3/3OB	3.286	7.992	1.87	3.50	this work
DFT/PW91	3.64	5.27	2.06	2.45	Piskorz *et al.*[1]
DFT/PBE+U	3.662	5.223	2.16	2.30	Puigdollers *et al.*[2]
DFT/PBE0	3.606	5.180	2.10	2.36	Evarestov *et al.*[3]

Table S9: Comparison of Structural Parameters of Cubic Zirconium Carbide ZrC

method	$a = b = c$ / Å	Zr-C / Å	Zr-Zr / Å	C-C / Å	source
DFTB3/3OB	4.954	2.112	3.505	2.158	this work
DFT/PBE	4.689	2.345	3.316	3.316	Xie *et al.*[4]
DFT/LDA	4.642	2.321	3.282	3.282	Khanzadeh *et al.*[5]
DFT/PBE	4.708	2.354	3.329	3.329	Khanzadeh *et al.*[5]
DFT/PBEsol	4.668	2.334	3.301	3.301	Khanzadeh *et al.*[5]

References

(1) Piskorz, W.; Gryboś, J.; Zasada, F.; Zapała, P.; Cristol, S.; Paul, J.-F.; Sojka, Z. Periodic DFT Study of the Tetragonal ZrO2 Nanocrystals: Equilibrium Morphology Modeling and Atomistic Surface Hydration Thermodynamics. *The Journal of Physical Chemistry C* **2012**, *116*, 19307–19320.

(2) Puigdollers, A. R.; Illas, F.; Pacchioni, G. Structure and Properties of Zirconia Nanoparticles from Density Functional Theory Calculations. *The Journal of Physical Chemistry C* **2016**, *120*, 4392–4402.

(3) Evarestov, R. A.; Kitaev, Y. E. New Insight on Cubic–Tetragonal–Monoclinic Phase Transitions in ZrO2: Ab Initio Study and Symmetry Analysis. *Journal of Applied Crystallography* **2016**, *49*, 1572–1578.

(4) Xie, C.; Oganov, A. R.; Li, D.; Debela, T. T.; Liu, N.; Dong, D.; Zeng, Q. Effects of Carbon Vacancies on the Structures, Mechanical Properties, and Chemical Bonding of Zirconium Carbides: A First-Principles Study. *Physical Chemistry Chemical Physics* **2016**, *18*, 12299–12306.

(5) Khanzadeh, M.; Alahyarizadeh, G. A DFT Study on Pressure Dependency of TiC and ZrC Properties: Interconnecting Elastic Constants, Thermodynamic, and Mechanical Properties. *Ceramics International* **2021**, *47*, 9990–10005.
